# Taxonomic and biosynthetic diversity of the marine actinomycete *Salinispora* across spatial scales

**DOI:** 10.1128/aem.02171-25

**Published:** 2025-12-08

**Authors:** Kaitlin E. Creamer, Gabriel Castro-Falcón, Ebru Ince, Victoria Vasilat, David Vereau Gorbitz, Alyssa M. Demko, Paul R. Jensen

**Affiliations:** 1 Center for Marine Biotechnology and Biomedicine, Scripps Institution of Oceanography, University of California San Diego 8784, La Jolla, California, USA; University of Delaware, Lewes, Delaware, USA

**Keywords:** *Salinispora*, diversity, natural products, biogeography

## Abstract

The spatial scales of bacterial taxonomic and natural product biosynthetic diversity remain poorly understood. This is especially true at the population level, where contrasts between small and large-scale biogeographical patterns are seldom reported. To address these unknowns for the marine actinomycete genus *Salinispora*, we sequenced the genomes of 99 strains cultured from sediments collected within a 1 m^2^ plot (microscale strains). Ninety-six of the microscale strains were identified as *S. arenicola*, suggesting that this is the most abundant species in the sediments sampled. These strains were assigned to 2 of the 11 populations identified based on 99% ANI among 61 public genomes obtained from 10 global collection sites (global strains). The populations showed evidence of geographic isolation, suggesting that barriers to dispersal or ecological contingencies limit distributions across large spatial scales. An assessment of *S. arenicola* biosynthetic gene diversity among 157 (combined microscale and global) genomes revealed 100 gene cluster families (GCFs), of which one-third were detected in either one or all strains. Sixty-seven percent of the global GCFs were detected among the microscale strains, indicating that deep sampling from a single location recovered a large percentage of the global biosynthetic diversity. Paired genomic and metabolomic analyses of the microscale strains linked compounds to an orphan PKS-NRPS GCF, while the metabolites ikarugamycin and fridamycin E were identified for the first time from *Salinispora*. This study provides insight into the diversity and biosynthetic potential of *Salinispora* at various spatial scales while expanding the collection of natural products reported from the genus.

**IMPORTANCE:**

The marine actinomycete genus *Salinispora* has become a model organism for natural product discovery and to address actinomycete diversity and distributions in marine systems. While biogeographic patterns have been reported at global scales, contrasts have yet to be made with the species diversity that can be recovered from a single location. Here we sequenced the genomes of 96 *S. arenicola* strains cultured from marine sediments collected within a 1 m^2^ plot and compared the diversity detected to public genomes obtained from global collection sites. The results provide evidence of geographic isolation among *S. arenicola* populations and biosynthetic genes that are mobilized across population boundaries. Multi-omic analyses linked compounds to their respective biosynthetic genes and revealed compounds not previously reported from the genus. This study adds to our growing understanding of *Salinispora* diversity and biosynthetic potential.

## INTRODUCTION

*Salinispora* was the first obligate marine actinomycete genus described (1–3). To date, hundreds of strains have been isolated from tropical and sub-tropical marine sediments, seaweed, and sponges collected around the globe (4–9). These slow-growing, Gram-positive bacteria in the family *Micromonosporeaceae* form branching mycelia (2) that extend over unknown spatial scales. The genus includes nine named species (3) and has been explored extensively as a source of novel natural products (10, 11). Compounds reported from this genus include salinosporamide A (12), a proteasome inhibitor that has entered clinical trials for the treatment of multiple myeloma and glioblastoma (13). To date, more than half of the compounds reported from this genus possess new chemical scaffolds, highlighting its potential as a source of novel natural products.

Among the first actinomycetes to have its genome sequenced, *Salinispora tropica* revealed exceptional biosynthetic potential, with *~*10% of the genome associated with natural product biosynthesis (14). Despite sharing 99% 16S rRNA gene sequence identity, *Salinispora* species exhibit remarkable biosynthetic diversity. Among 118 sediment-derived strains collected from global locations, 305 biosynthetic gene cluster families (GCFs) were detected, indicating a vast reservoir of biosynthetic potential relative to the 31 natural product families chemically characterized from the genus (15). Over half of this diversity comes from biosynthetic gene clusters (BGCs) that were observed in only one or two strains and were likely acquired through horizontal gene transfer (HGT). In support of this, many *Salinispora* BGCs are located within highly variable genomic islands, suggesting a “plug and play” mechanism of acquisition and selection (15, 16). In contrast, some *Salinispora* natural products have been described as species-defining traits (17) with the associated BGCs revealing strong phylogenetic signals that are congruent with the species tree. These observations support the concept that select natural products represent ecotype-defining traits (18, 19).

Much has been learned about the biogeographic patterns of marine bacteria from large-scale studies (20, 21). Yet, relatively little is known about the forces that shape bacterial distributions in nature (22). One theory is that microbial distributions are governed by environmental selection as opposed to dispersal limitations (23). In this scenario, a species should be present in all environments capable of supporting its growth. The biogeographic distribution of the genus *Salinispora* varies by species, with *S. arenicola* being the most broadly distributed (24). In contrast, *S. pacifica* has mainly been reported from the Pacific Ocean, but not the Caribbean Sea, while *S. tropica* has only been reported from the Caribbean Sea (25). While these patterns have emerged, no attempts have been made to deeply sample a single location to assess *Salinispora* species diversity.

This study aimed to assess the taxonomic and biosynthetic gene diversity of *Salinispora* strains cultured from marine sediments collected within a one square meter quadrat. Given that *Salinispora* produces vegetative hyphae, we also tested for clonality among the spatially confined strains to provide context for growth in marine sediments. We compared these results with those previously reported from 10 global collection sites to determine how a highly localized biodiversity estimate reflects geographically broader patterns and to explore linkages between populations and their functional traits in the context of specialized metabolism.

## RESULTS AND DISCUSSION

### *Salinispora* isolation and genome sequencing

Sixteen sediment samples collected within a 1 m^2^ 4 × 4 grid placed near a coral reef in Fiji were processed for the selective isolation of actinomycetes. Over 200 strains with *Salinispora*-like morphologies (2) were isolated in pure culture. All strains were tested for the requirement of seawater for growth, a hallmark of the genus (1). 16S rRNA sequencing of the seawater-requiring strains identified 172 as *S. arenicola*, the vast majority of which had identical 16S sequences that could be assigned to the “standard” (ST) sequence type (24). The only four strains with different 16S sequences were tentatively identified as *S. pacifica*. These results suggest that *S. arenicola* is the most abundant *Salinispora* species present in the sediments sampled, which is concordant with its broad geographic distribution (24). This suggestion is further supported by the only culture-independent study to assess *Salinispora* diversity, which assigned 82% of 45 cloned sequences to *S. arenicola* (26). The high levels of 16S sequence identity among *Salinispora* species have made it difficult to assess relative species abundances using short read, next-generation amplicon sequencing.

To obtain more information about the genetic diversity among the *S. arenicola* strains isolated from the 1 m^2^ quadrat, six strains were selected for genome sequencing from each of the 16 sediment samples (96 strains in total). The 96 strains, herein referred to as “microscale strains,” were morphologically diverse, with some producing white aerial hyphae that preceded the formation of black spores (Fig. S1). Colony morphologies ranged from “popcorn-like” to displaying circular concentric growth, with some showing extensive vertical growth on the agar surface. Colony pigmentation varied from pale yellow to deep orange. This morphological diversity led us to believe that the strains were genetically diverse, despite having identical 16S rRNA sequences. In addition, genomes were obtained for three of the *Salinispora* strains that were tentatively identified as *S. pacifica*.

The genome assemblies averaged 88 contigs with an *N*_50_ of 185,576 bp, a largest contig length of 470,884 bp, and an average genome size of 5.6 Mb. The GC content averaged 69.62% with 5,059 total genes, three rRNA genes, and 64 tRNA genes. CheckM (27) estimates indicated that the genome assemblies were 99–100% complete with 314 *Actinomycetales* marker genes present and 0.19% contamination. Assembly statistics and NCBI accession numbers are shown in Table S1. Based on 95% average nucleotide identity (ANI) values across all 99 strains and the nine type strains for the genus, 96 strains were confirmed as *S. arenicola*, one as *S. pacifica*, and two as *S. oceanensis*. The 96 microscale *S. arenicola* genomes had an average ANI of 99.02% (range: 98.4847–99.9974%; SD = 0.35%) with no evidence of clonality (100% ANI). From the 4,560 unique pairwise comparisons among the 96 microscale genomes, 6 pairs (12 genomes) shared ≥99.99% ANI, 265 pairs (84 genomes) shared ≥99.6% ANI, and all 4,560 pairs shared ≥96% ANI. These values indicate that the population recovered is comprised of different but closely related *S. arenicola* strains (28) as opposed to a clonal mycelial expansion. Among the 12 genomes that shared ≥99.99% ANI, only one pair was isolated from the same sediment sample and both originated from different isolation plates. Overall, only 39 of the 4,560 pairwise genome comparisons (0.86%) included strains recovered from the same isolation plate, and only 4 of these shared ≥99.6% ANI. As such, the levels of sequence identity were not highly influenced by the isolation of strains from the same agar plate, while the targeting of colonies with different morphologies likely maximized the diversity detected. Given that most of the microscale strains were *S. arenicola*, we focused on this species for the remainder of the study.

### *S. arenicola* diversity across spatial scales

**Fig 1:**
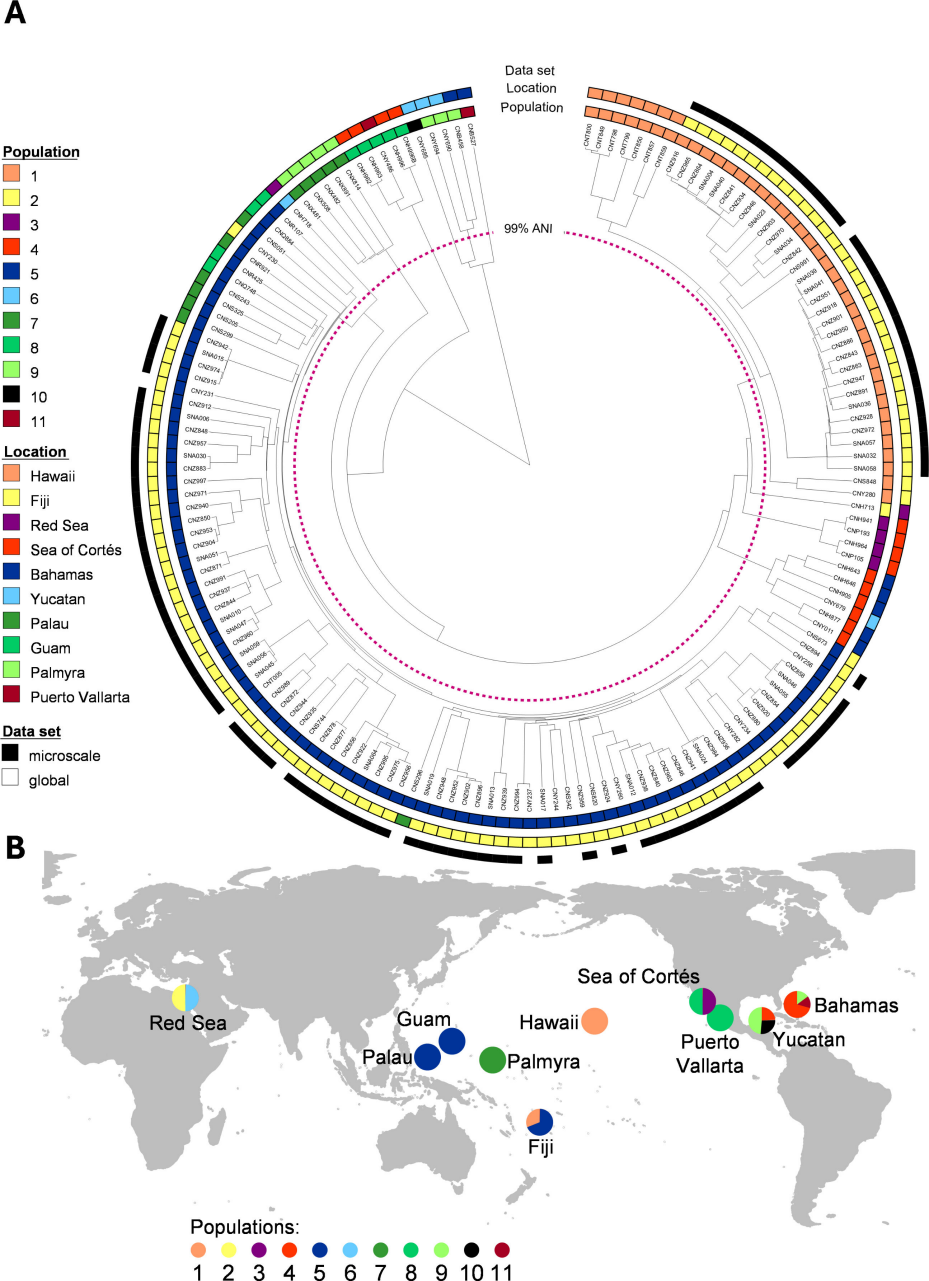
ANI dendrogram and sources of *S. arenicola* strains. (**A**) The inner purple line demarcates 11 color-coded 99% ANI populations. Strain origin (location) is indicated by the second color-coded circle. The outer circle (black) indicates the microscale strains. (**B**) Geographic distributions of the 11 (99%) ANI populations. Pie graphs indicate the proportion of each population isolated from that location. The map was made using the maps package in RStudio.

We next assessed the diversity of the 96 *S*. *arenicola* microscale strains relative to 61 publicly available genomes sourced from the following 10 global locations (number of genomes from each location in parentheses): the Bahamas (7), the Yucatan (4), Puerto Vallarta (1), the Sea of Cortés (8), Hawaii (7), Palmyra (5), Fiji (16), Guam (4), Palau (7), and the Red Sea (2). A 99% ANI dendrogram of the combined 157 *S*. *arenicola* strains revealed 11 populations (Fig. 1A), of which the microscale strains were assigned to populations 1 (30 strains) and 5 (66 strains). A phylogenomic tree constructed using 324 single-copy conserved genes delineated many of the ANI populations with some exceptions that appear to be location dependent (Fig. S2). The high level of sequence similarity among the strains makes it difficult to assess evolutionary relationships without accounting for recombination (24). Interestingly, the 16 Fijian strains in the global collection, which were isolated between 2004 and 2011 from marine sediments collected throughout Fiji, belong to the same two ANI populations (1 and 5) as the microscale strains isolated in 2017. This finding reveals the temporal stability of *S. arenicola* populations 1 and 5 over a 13-year time span and suggests they are the only two populations at this location.

There was evidence of geographic isolation among the 11 *S*. *arenicola* ANI populations, with five reported from only one location and five locations yielding only one population (Fig. 1B). While only two populations were recovered from Fiji, neither is exclusive to this location, with seven population 1 strains identified from Hawaii and 11 population 5 strains identified from Palau and Guam. Given the current data set, ANI populations 1 and 5 appear to be endemic to the Central and Western Pacific. The five strains from Palmyra Atoll, which is also in the Central Pacific, are the only strains in population 7 suggesting that the geographic isolation of this location facilitated genetic divergence. The remaining eight populations all show varying levels of geographic patterning, with populations 3 and 8 recovered exclusively from the Pacific coast of Mexico (Sea of Cortés and Puerto Vallarta), populations 4, 9, 10, and 11 recovered exclusively from the Caribbean (Yucatán and Bahamas), and populations 2 and 6 recovered exclusively from the Red Sea. These results, which are based on all currently available *Salinispora* genomes, amplify the relationships between geography and fine-scale *S. arenicola* diversity (24). Nonetheless, the uneven number of genomes available across the global sites likely impacts these findings, which will be further informed as more genome sequences become available.

### Biosynthetic potential across spatial scales

We next assessed the biosynthetic potential of the 157 (combined microscale and global) *S. arenicola* strains. BGCs were detected using antiSMASH v7.0 (29) and grouped into 204 GCFs using BiG-SCAPE (30). Manual inspection revealed an overestimation of the GCF total likely due to the splitting of BGCs onto different contigs. Manual curation, facilitated by 16 experimentally validated *S. arenicola* BGCs (15) and the comparison tools antiSMASH and clinker (31), allowed us to reduce the number of GCFs to 100 (Fig. S3; Data S1) across eight biosynthetic classes. This finding emphasizes the value of manual curation and the potential for automated tools to overestimate biosynthetic richness.

**Fig 2:**
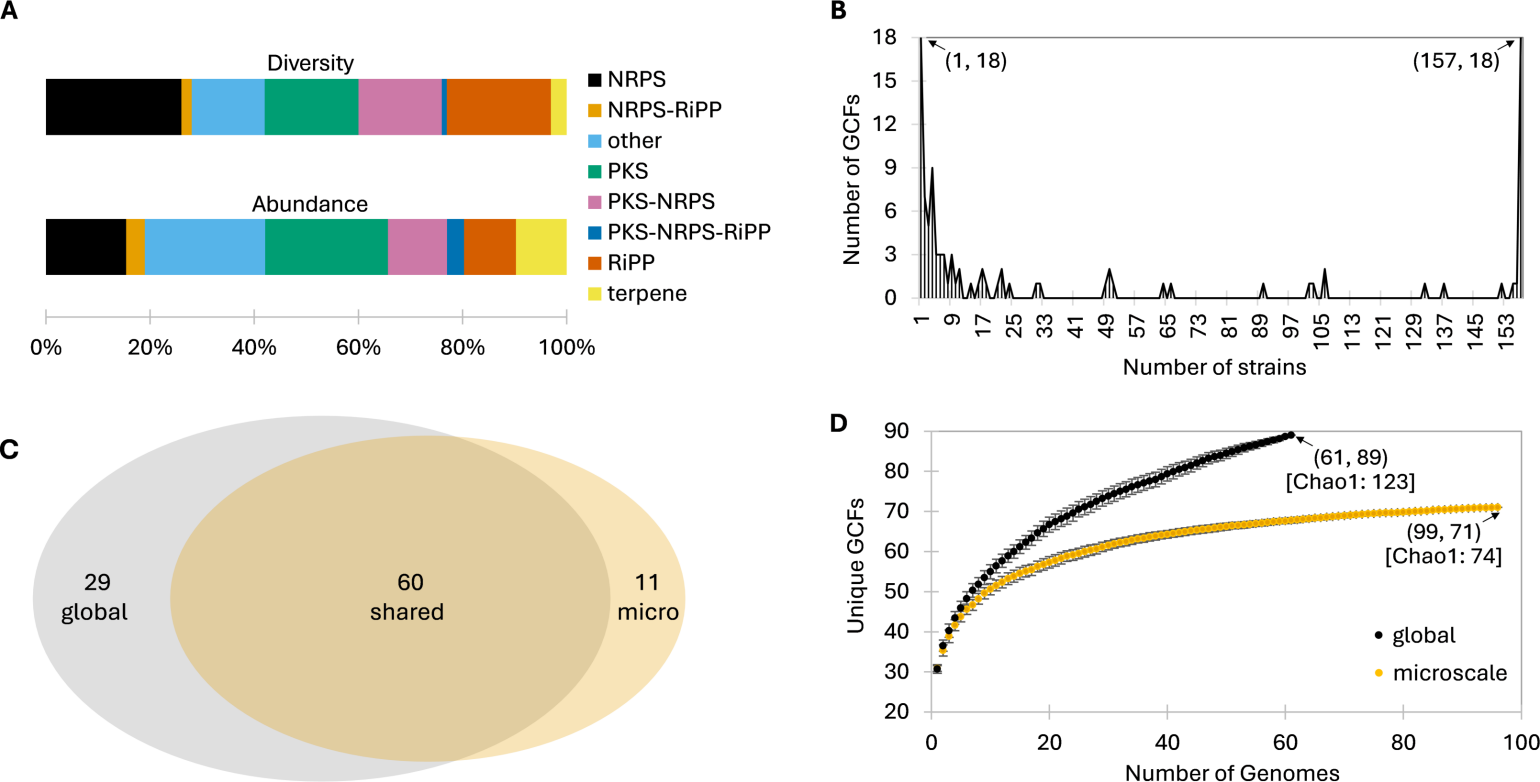
*S. arenicola* biosynthetic potential. (**A**) Relative diversity and abundance of biosynthetic classes across 157 *S*. *arenicola* genomes. Diversity is expressed as the percentage of all GCFs in each biosynthetic class. Abundance is expressed as the percentage of all BGCs in each biosynthetic class. (**B**) Number of strains in which each GCF was observed. Eighteen GCFs were observed in only one strain, while another 18 were observed in all 157 strains. (**C**) GCF distributions between the microscale and global genomes. (**D**) GCF rarefaction curves for the microscale and global genomes. Average *y*-axis values (black or orange circles) and standard deviation are plotted. Numbers in parentheses indicate total number of genomes followed by total number of GCFs. Numbers in brackets indicate Chao1 diversity estimates.

The most diverse classes of natural product genes detected were annotated as non-ribosomal peptide synthetases (NRPSs) and ribosomally synthesized and post-translationally modified peptides (RiPPs), which accounted for 26 and 20 GCFs, respectively (Fig. 2A). In contrast, the most abundant BGC types were polyketide synthases (PKSs) (1,140) followed by “others” (1,117), representing 23.6% and 23.1% of the total, respectively. The distribution of GCFs across strains revealed an interesting pattern, with maxima (18 in both cases) represented by either singletons (GCFs observed in only one strain) or core GCFs (GCFs observed in all 157 strains) (Fig. 2B). These maxima accounted for 36% of all GCFs, suggesting that either recent acquisitions or strong positive selection accounts for a large percentage of the biosynthetic diversity observed. GCFs that were only observed in one (singleton) or two (doubleton) genomes accounted for 25% of the *S. arenicola* GCF diversity.

A comparison of biosynthetic diversity revealed that 60 of 89 GCFs detected among the global strains were also detected among the microscale strains. This demonstrates that deep sampling from surface sediments within a 1 m^2^ quadrat yielded most of the biosynthetic diversity obtained from the global locations (Fig. 2C). The percentage of shared GCFs did not change after excluding the 16 global strains sourced from Fiji (the microscale strain country of origin). Furthermore, 11 GCFs were only observed among the microscale strains supporting the value of deep sampling from a spatially confined area. Nonetheless, plateauing in the microscale strain GCF rarefaction curve indicates that continued genome sequencing would yield little additional diversity (Fig. 2D). In contrast, the Chao1 diversity estimate for the globally sourced strains suggests that additional sequencing would continue to yield new GCFs, which would likely impact the number of singleton and shared GCFs reported here.

**Fig 3:**
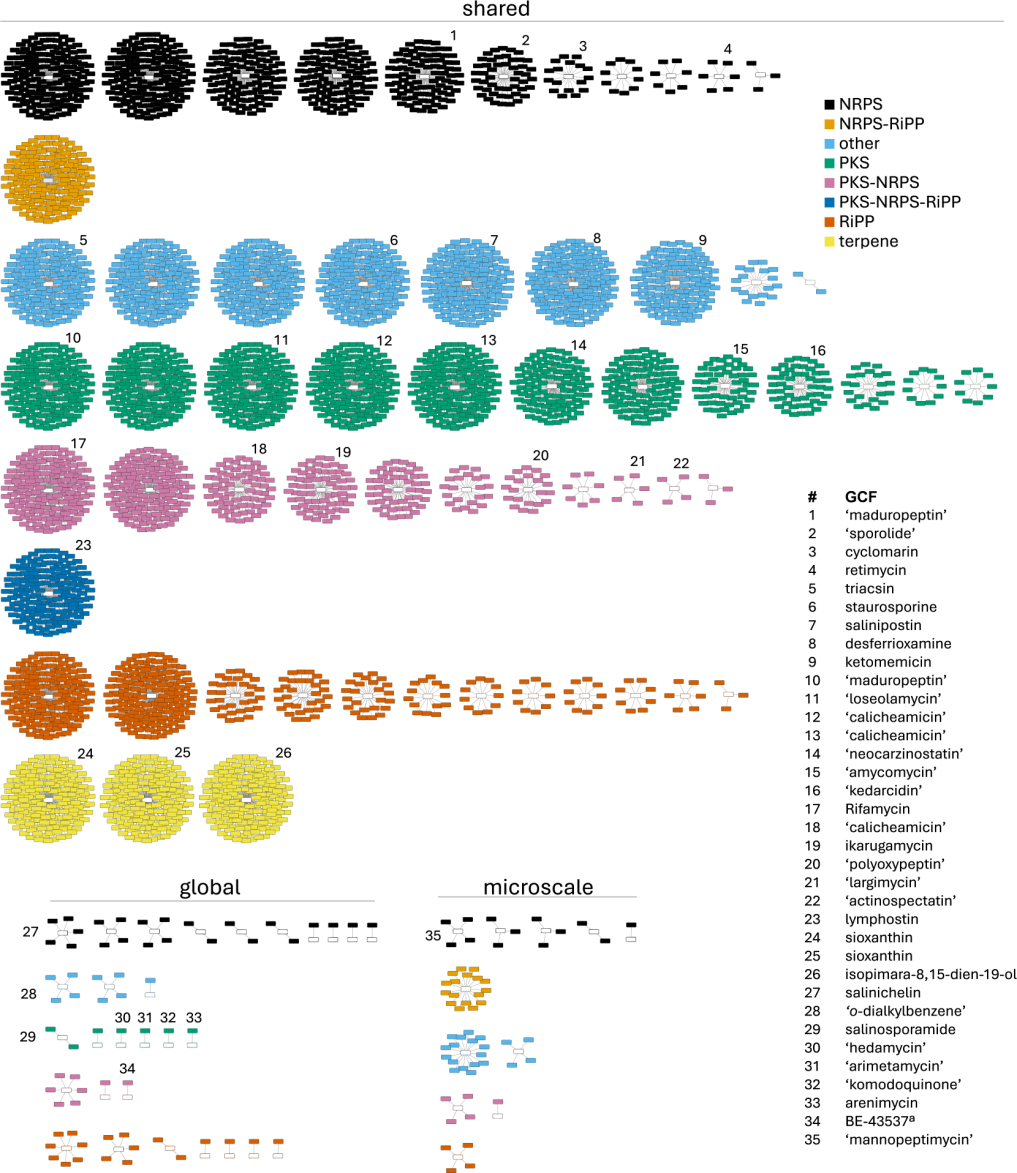
*S. arenicola* gene cluster family (GCF) network. Each node represents a BGC and each cluster of nodes a GCF. GCFs are categorized as shared (present in both data sets), global (global-specific), or microscale (microscale-specific) and color-coded by biosynthetic class. Clusters are composed of *n* + 1 nodes (where *n* = number of BGCs). As such, singletons are represented by two nodes. GCF annotations are provided for experimentally validated *Salinispora* products or MIBiG BGC matches (in quotations).

A GCF network was created to visualize the 100 GCFs along with their biosynthetic class, annotations, and representation among the microscale and global *S. arenicola* strains (Fig. 3). Benefiting from prior research (11), it was possible to confidently assign 17 of these GCFs to 16 compound families, with the sioxanthin BGC divided between two GCFs in accordance with its non-clustered distribution (32). Because most GCFs (83%) have not been experimentally linked to a metabolite, we used the “known cluster similarity” tool in antiSMASH to provide additional annotations (33). This revealed eight GCFs with high similarity scores to the MIBiG BGCs encoding arimetamycin (1 BGC, 91%), hedamycin (1 BGC, 87%), komodoquinone (1 BGC, 68%), largimycin (5 BGCs, 65%), polyoxypeptin (21 BGCs, 59%), mannopeptimycin (3 BGCs, 59%), loseolamycin (157 BGCs, 56%), and actinospectacin (4 BGCs, 52%), suggesting that similar metabolites might be produced by *S. arenicola* (Fig. S4). Of these, the mannopeptimycin BGC was only observed in the microscale strains. Interestingly, the loseolamycin-like BGC was observed in all strains, yet the product has yet to be reported from *Salinispora*.

The BGCs associated with nine additional “orphan” GCFs showed moderately high similarity scores (12–40%) to enediyne BGCs in the MIBiG database (Fig. S5). Some of these matches appeared to be split into multiple GCFs. For example, two GCFs appear to represent two halves of the calicheamicin BGC. Three additional orphan GCFs in *S. arenicola* showed low, yet meaningful, similarity scores to experimentally characterized iterative type I and type II PKS BGCs (Fig. S6). One of the type II PKSs is found in all strains and is thought to produce the black spore pigment (14). When these GCFs are included, the annotated GCFs in the *S. arenicola* genomes increase to 36%, a relatively high level for bacterial genomes and a reflection of the extensive effort that has gone into the discovery of natural products from this genus.

### GCF distributions

**Fig 4:**
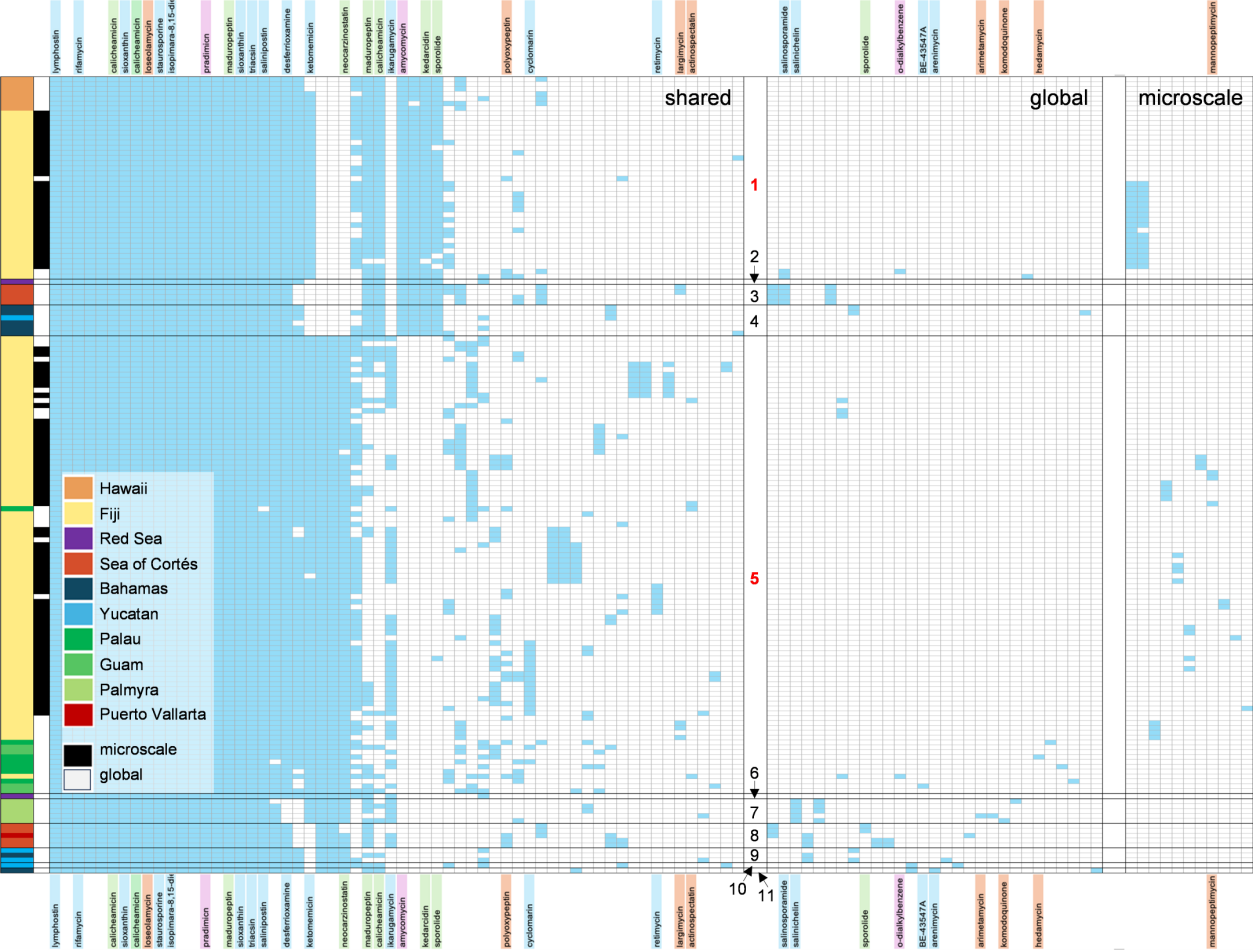
GCF distributions in *S. arenicola*. Columns indicate presence (blue) of 100 GCFs across 157 *S*. *arenicola* genomes (*y*-axis). The three matrices describe shared, global-specific, or microscale-specific GCFs. For each matrix, the columns are arranged from left to right according to GCF abundance. Annotated GCFs are indicated by compound names and highlighted according to confidence level (blue = validated, orange = 56–91% MIBiG match, green = 12–40% MIBiG match to enediyne BGCs, and pink = select MIBiG matches with 25–32% similarity). The first column of the *y*-axis is colored according to geographic origin and ordered by 99% ANI populations (Fig. 1), which are numbered (microscale shown in bold red) and demarcated by gray horizontal lines. The second column delineates microscale (black) and global strains (white). See Data S2 for strain designations.

We next assessed GCF distributions by mapping them to the 11 populations distinguished by 99% ANI (Fig. 4). As previously reported, the rifamycin and lymphostin BGCs remain conserved at the species level (15), the contiguous salinispostin BGC is in all but one *S*. *arenicola* strain (34), and the functionally equivalent exchange of the desferrioxamine and salinichelin siderophore BGCs (35) remains intact among the new genomes. While the species specificity of *Salinispora* natural products has been discussed (15), we see evidence of population-level specificity in two enediyne GCFs and the amycomycin GCF (Fig. 4), which are highly conserved in populations 1–4 but largely absent in populations 5–11. Conversely, an enediyne and two unannotated GCFs were highly conserved in populations 5–11 but absent in populations 1–4. These two population groups (1–4 and 5–11) formed well-separated clusters in both non-metric multidimensional scaling (NMDS) and heatmap plots based on Jaccard distance similarities of GCF distributions (Fig. S7 and S8), with PERMANOVA analysis indicating that both population (*R*^2^ = 0.67, *P* < 0.01) and location (*R*^2^ = 0.26, *P* < 0.01) were significant. Other notable GCF distributions include the ketomemicin GCF (absent from populations 2–4 and 8–11) and the ikarugamycin GCF (absent from populations 1–4 and 9–11). The cyclomarin, retimycin, salinosporamide, and salinichelin GCFs were much less common yet also showed population-level specificity. Conversely, the polyoxypeptin GCF, along with some GCFs that could not be annotated, appears to be randomly distributed among the populations.

### Microscale strain metabolomes

**Fig 5:**
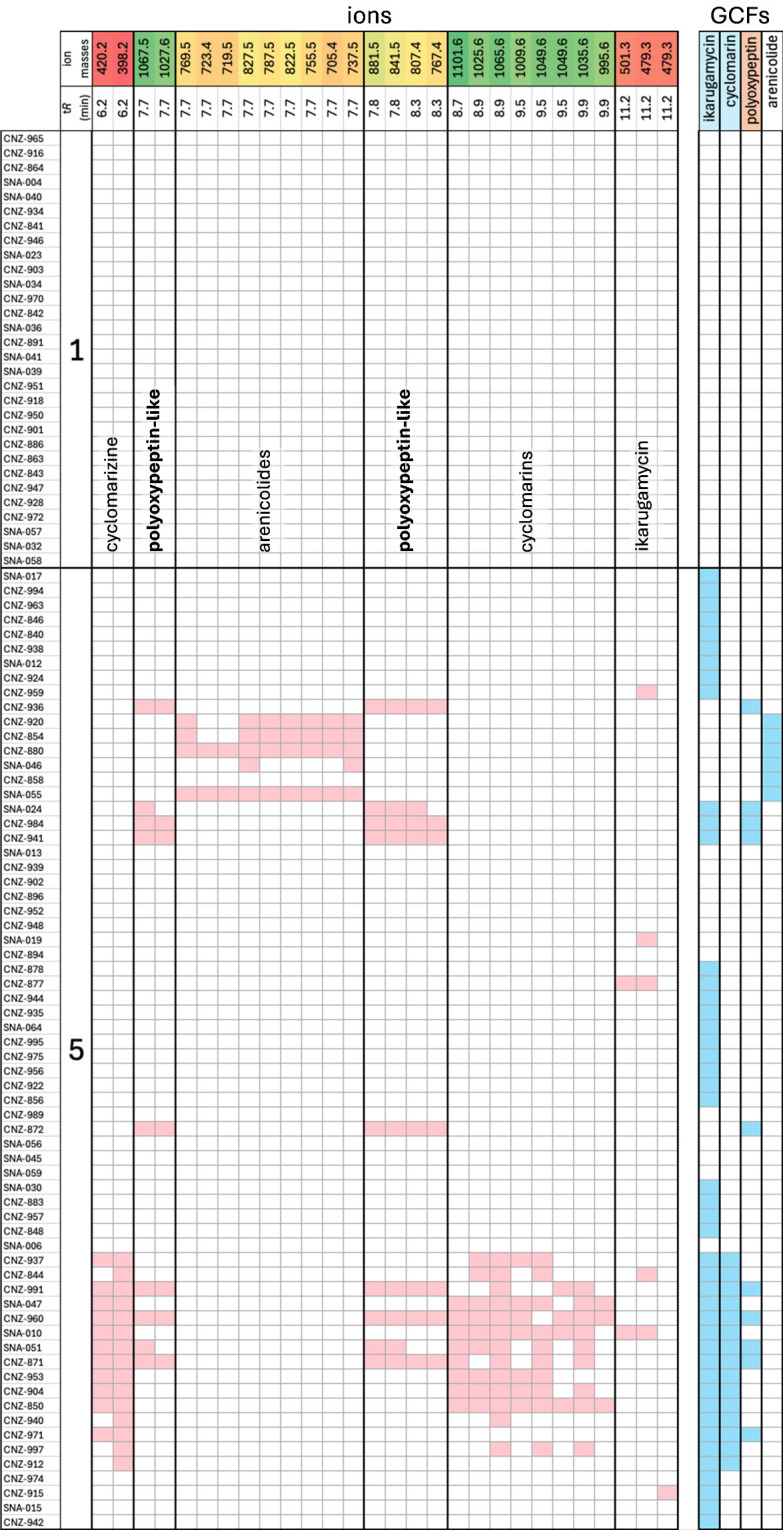
Paired omic analysis of microscale strains. Presence-absence table of select ions linked to validated or putative GCFs (see Data S3 for full data set). Columns represent ions (with red, yellow, and green gradients representing molecular weight from light to heavy) ordered by retention times (*t*_R_). Compound names linked to ions are listed. Rows delineated into microscale strain populations 1 and 5 based on 99% ANI groupings. Ions and GCFs shown in this figure (colored boxes) were only observed in population 5. Pink: ion detected, Blue: GCF present.

We next compared the metabolomes of the two *S*. *arenicola* microscale populations (1 and 5) using LC-UV-MS. We also used paired-omics (36) to search for linkages between metabolite production and orphan BGC distributions. We detected ikarugamycin in extracts of 5 of the 45 strains in which the BGC was observed (Fig. 5). This supports prior observations that similar *Salinispora* BGCs are not equally expressed (37) and emphasizes the value of having multiple strains containing similar BGCs when searching for their small molecule product(s). This is the first report of ikarugamycin production in *Salinispora* cultures, although production was linked to the BGC by heterologous expression (38). Cyclomarins A, B, and D, and the shunt product cyclomarizine, were also detected with production patterns largely congruent with GCF distributions. Arenicolides were detected but have yet to be experimentally linked to a BGC. Here, we found a near-perfect match between arenicolide production and an orphan type I PKS BGC in five of the six strains. The starting and extension modules of this BGC match what is expected for arenicolide biosynthesis, yet it does not appear to be fully assembled, as is commonly observed in modular PKS genes (14). We also linked the production of unknown ions from population 5 to a GCF with high (59%) similarity to the polyoxypeptin BGC (39), suggesting they may be related natural products (also referred to as the “azinothricin family” of compounds) (Fig. 5). These ions (*m/z* 1,067.6 and 1,027.6, 881.5 and 841.5, 807.4, and 767.4) were detected in nine strains and could be attributed to three compounds with identical or similar predicted molecular formulas to the azinothricin family of natural products (Fig. S9).

We also analyzed the metabolomes of one *S*. *pacifica* and two *S*. *oceanensis* strains isolated as part of this study. Notably, the anthraquinone fridamycin E is reported for the first time from the genus *Salinispora*. This compound is the product of an angucycline type II PKS BGC, such as those coding for the grincamycins and tetrangomycins (40, 41). The producing strain, *S. oceanensis* CNZ-875, encodes three type II PKS BGCs, two of which contain KS domains predicted by NaPDoS2 to produce angucyclines (42). However, only one of these, the putative fridamycin BGC, includes the cyclase, aromatase, and oxidoreductase functions that are characteristic of angucycline biosynthesis (Fig. S10 through S12). A broader analysis of available *Salinispora* genomes led to the detection of the candidate fridamycin BGC in two additional *S. oceanensis* strains (CNT-124 and CNT-584) and two *S*. *fenicalii* strains (CNR-942 and CNT-569) (Fig. S13).

### Conclusions

The genome sequences of 99 *Salinispora* strains cultured from a 1 m^2^ quadrat (microscale strains) deployed near a coral reef in Fiji revealed no evidence of clonal, mycelium expansion at this spatial scale. The 96 strains identified as *S. arenicola* belonged to 2 (populations 1 and 5) of the 11 populations based on 99% ANI detected among 61 strains isolated from 10 global locations. Based on this culture-dependent analysis, barriers to dispersal or ecological contingencies appear to limit the distribution of *S. arenicola* populations across large spatial scales. This is supported by evidence of geographic isolation, with populations 1 and 5 limited to the Pacific and population 7 only reported from the remote Pacific atoll Palmyra, among other patterns. These results complement prior evidence of both sub-species and species-level *Salinispora* biogeographical distributions (24). Nonetheless, the patterns observed here, which are based on all currently available genome sequences, will likely change as additional genome sequences become available.

Extensive prior work linking *Salinispora* natural products to their respective BGCs made it possible to reduce the automated assessment of biosynthetic diversity from 204 to 100 GCFs, in most cases due to BGCs being split onto multiple contigs. These results emphasize the value of manual annotations and support prior observations that automated assessments can result in an overestimation of biosynthetic potential (43). It’s noteworthy that the microscale strains accounted for only 18% of the population level diversity yet included 67% of the biosynthetic diversity observed among the global strains. While this may reflect differences in the granularity of the analyses (i.e., 61 global strains compared to 96 microscale strains), HGT may also play a role (19). This is supported by the 18 GCFs that were observed in only one strain (Fig. 2B) and may have been recently acquired (Fig. 4). High rates of BGC acquisition relative to population diversification could account for these patterns. Nonetheless, the two microscale populations (1 and 5) clearly separate based on GCF distributions in an NMDS plot (Fig. S7), suggesting these functional traits may be associated with population diversification among co-occurring strains. Finally, the large data set made it possible to link several compounds, including the azinothricins, to candidate GCFs. This study provides insight into the spatial scales of bacterial taxonomic and biosynthetic diversity and the value of paired genomic-metabolomic analyses in natural product research.

## MATERIALS AND METHODS

### Sediment collection and *Salinispora* isolation

Sixteen sediment samples were collected as previously described (44) around Nacula Island, Fiji, via SCUBA in June 2017 from a 1 m^2^ quadrat evenly divided into 16 (4 × 4) sections. The quadrat was placed in an area of coarse calcareous sediment next to a reef (depth: 10 m; coordinates: 16°53.578′S, 177°23.076′E). Sediment from each of the 16 sections was collected in Whirl-pak (Nasco) bags and frozen (−20°C) until processing.

To selectively culture *Salinispora*, frozen sediments (ca. 1 g) from each of the 16 sub-quadrat samples were placed in sterile petri dishes and dried (3 days) in a laminar flow hood. Sterile cylindrical sponges wetted with sterile seawater were used to stamp sediment onto petri plates containing: (i) A1 (10 g/L starch, 4 g/L yeast extract, 2 g/L peptone, 22 g/L Instant Ocean, 16 g/L agar, 1 L diH_2_O, and cycloheximide added after autoclaving at a final concentration of 100 µg/mL) and (ii) SWA (22 g/L Instant Ocean, 16 g/L agar, 1 L diH_2_O, and cycloheximide added after autoclaving at a final concentration of 100 µg/mL). Stamping was performed in a spiral pattern with ca. 11 stamps per plate. Four plates each of A1 and SWA were stamped per sample (128 plates total). Plates were incubated at room temperature and monitored for >2 months.

A total of 229 single colonies with *Salinispora*-like morphologies were isolated by re-streaking onto new plates of the same medium. Seawater growth assays were performed using split-petri plates with A1 agar on one side and A1 agar in which DI water replaced seawater on the other. Colony PCR using FC127 (5′-AGAGTTTGATCCTGGCTCAG-3′) and RC1492 (5′-TACGGCTACCTTGTTACGACTT-3′) 16S rRNA gene primers was performed on strains (*n* = 176) that failed to grow without saltwater. Strains identified as *Salinispora* based on 16S sequencing were cryopreserved (−80°C) in A1 plus 10% glycerol from each of the 16 sediment samples. To represent both genetic and phenotypic diversity, six strains from each sub-quadrat were genome-sequenced, including all unique 16S rRNA sequence types and maximizing variation in isolation source (plate number, culturing medium) and morphology. As a result, some sequenced isolates originated from the same original isolation plate.

### Cultivation and sample preparation

*Salinispora* strains were grown from frozen stocks in A1 liquid media (60 mL) at 28°C at 230 rpm shaking for 1–2 weeks with glass beads to prevent clumping. Cultures were subsampled for DNA extraction (4 × 1 mL of culture centrifuged to obtain cell pellets, stored at −80°C) and metabolomic analysis (14 mL, stored at −20°C).

### Genome sequencing and assembly

The Wizard Genomic DNA Purification Kit (Promega) was used for genomic DNA extractions with modifications for Gram-positive cells including the addition of freshly prepared lysozyme (10 mg/mL, Sigma Aldrich) and the use of wide-bore pipette tips to prevent shearing. Extracted gDNA was quantified with a NanoDrop 1000 spectrophotometer (Thermo Fisher Scientific) and a Qubit 3.0 fluorometer (Thermo Fisher Scientific), while quality was assessed by gel electrophoresis. Genomic DNA was sequenced at two facilities: (i) UC Davis, Illumina MiSeq, PE300, 700 bp inserts; resulting in ~17.7 million reads with ~11.7% PhiX spike-in, and an overall Q30 >60%; and (ii) UCSD IGM, Illumina NovaSeq 6000, S4 PE150, 400 bp inserts prepared with a Nextera XT library kit (Illumina) and a pre-pooling MiSeq check run; resulting in 5–27 million reads per genome, and an overall *Q*_30_ > 90% for each genome.

Bioinformatic analyses were performed on the Triton Shared Computing Cluster at the San Diego Supercomputer Center (https://www.sdsc.edu/systems/tscc/index.html). Raw MiSeq and NovaSeq data sets were assembled separately. Genome assembly and annotation were performed using bactopia version 2.0.3 (45) and a Nextflow-enabled (version 22.04.0) nf-core workflow. See General Experimental in the supplemental material for details.

### ANI

Fastani version 1.32 (46) within bactopia (45) was used to calculate ANI values, which were visualized as a dendrogram using the package bactaxR (https://github.com/lmc297/bactaxR) (47) and custom scripts using packages reshape2 and ggtree (48, 49) in RStudio. A phylogenetic tree of conserved single-copy core genes for the microscale (99 genomes), global *S. arenicola* (61 genomes), and combined (157 genomes) genomes was calculated using PhyloPhlAn 2.0 (50). Subsequent phylogenetic trees of all concatenated marker genes were calculated with a RAxML with PROTCATLG model of evolution with 100 bootstraps (51) and visualized with iTOL (52).

### GCF analysis

*S. arenicola* genomes were analyzed with antiSMASH 7.0 (29) to detect BGCs, which were then analyzed using BiG-SCAPE to calculate similarities and generate GCFs (30). BiG-SCAPE networks were visualized in Cytoscape (53). AntiSMASH and clinker (31) were used to analyze and manually refine the BiG-SCAPE GCFs.

For manual GCF analyses, 16 *Salinispora* BGCs that have been experimentally linked to their cognate natural products (e.g., by gene knock out or heterologous expression) were assigned to GCFs. AntiSMASH and clinker (31) were then used to compare (i) the BGCs within those same GCFs and (ii) the BGCs within different GCFs within the same biosynthetic class. These comparisons were performed on random subsets of up to 20 BGCs per GCF until no outgroups were detected. We also removed forty-four NRPS or type I PKS GCFs that contained BGCs of <15,000 bp and were deemed incomplete. The triacsin GCF (54) was manually added as it was not detected by antiSMASH version 7, and nine additional GCFs were added by breaking up large BGCs identified by antiSMASH. A table summarizing the BGCs detected in *S. arenicola* strains and their GCF and biosynthetic class assignments was imported into Cytoscape (53) and was used to create, color, and annotate the gene cluster network. GCF presence-absence tables were analyzed using an R script to determine the number of new GCFs added with each new genome sequence. This was repeated 100 times, with randomized strain order, to produce averages and standard deviations. A Jaccard index dissimilarity matrix was computed based on a GCF presence-absence table considering all microscale and global strains. For NMDS, dimensionality (*k*) was set to three, maximum number of random starts (*trymax*) was set to 500, and maximum number of iterations (*maxit*) was set to 500.

### Metabolomic analyses

Aliquots (8 mL) of *Salinispora* cultures were extracted with EtOAc (1:1 vol:vol) by vigorous shaking in capped test tubes. Test tubes were centrifuged to separate aqueous and organic phases, after which the organic phase was transferred to a clean test tube and dried in a speed-vac. Extracts were resuspended in methanol (200 µL) and analyzed on an Agilent 1100 Series HP system with UV and ELS detection and also on an analytical Agilent 1260 Infinity Series LC system coupled to a 6530 Series Q-TOF mass spectrometer, both using a C18 Phenomenex Luna column (5 µm, 100 mm × 4.6 mm) with a 10 min solvent gradient from 10% to 100% MeCN (0.1% FA) in water (0.1% FA) with 1.0 mL min^−1^ flow rate. LCMS data were converted to mzxml format and imported into MZmine v2 (55) for processing (see General Experimental in the supplemental material for details). The resulting aligned peak list was exported as a csv table that included detected masses, retention times, and peak heights.

## Data Availability

Genomic data for microscale strains are available from the National Center for Biotechnology Information (NCBI), with accession numbers provided in Table S1. Genomic data for global strains are available from the U.S. Department of Energy Joint Genome Institute Integrated Microbial Genomes (IMG) database. LC-MS/MS data are publicly available in the MassIVE data repository (http://massive.ucsd.edu, MSV000098188). Data S1 through S3 contain a list of detected BGCs and their respective GCF assignments, a presence-absence table of GCF distribution in microscale strains, and a presence-absence table of LCMS ions in microscale metabolomes, respectively.

## SUPPLEMENTAL MATERIAL

Data S1 aem.02171-25-s0001.xlsx Complete list of biosynthetic gene clusters (BGCs) associated with the 100 gene cluster families (GCFs),

Data S2 aem.02171-25-s0002.xlsx BGC distributions in strains.

Data S3 aem.02171-25-s0003.xlsx Paired omics.

Supplemental material aem.02171-25-s0004.pdf Supplemental methods, Table S1, and Figures S1 to S13.

ASM does not own the copyrights to Supplemental Material that may be linked to, or accessed through, an article. The authors have granted ASM a non-exclusive, world-wide license to publish the Supplemental Material files. Please contact the corresponding author directly for reuse.
