# Supplementary material for: Taxonomic and biosynthetic diversity of the marine actinomycete Salinispora across spatial scales
Source: Appl Environ Microbiol. 2025 Dec 8;92(1):e02171-25. doi: 10.1128/aem.02171-25 (PMC12838356; doi:10.1128/aem.02171-25)

Supplementary Information

Taxonomic and biosynthetic diversity of the marine actinomycete *Salinispora* across spatial scales

Kaitlin E. Creamer[1,2,3,#], Gabriel Castro-Falcón[1,#], Ebru Ince[1,4], Victoria Vasilat[1], David Vereau Gorbitz[1,5], Alyssa M. Demko[1], Paul R. Jensen[1*]

[1]Center for Marine Biotechnology and Biomedicine, Scripps Institution of Oceanography, University of California San Diego, La Jolla, CA 92093, USA

[#] Authors contributed equally to this work.

* Email: pjensen@ucsd.edu

Present addresses:

[2] Innovative Genomics Institute, University of California, Berkeley, 2151 Berkeley Way, Berkeley, CA 94720, USA

[3] Department of Earth and Planetary Science, University of California, Berkeley, 307 McCone Hall Berkeley, California, 94720 USA

[4] Department of Biology, Faculty of Science, Dicle University, Diyarbakır, Turkey

[5] School of Molecular and Cellular Biology, University of Illinois at Urbana Champaign, Urbana, Illinois, USA

General Experimental……………………………………………………………………………………1
Figure S1. Morphologies of microscale *Salinispora* strains grown on agar……………………………...……3
Table S1. Microscale genome statistics and accession numbers…..…..……………………..……………4
Figure S2. Phylogeny of microscale *Salinispora* strains…………………………………………………7
Figure S3. Manual assessment of BiG-SCAPE GCFs ………………………………………….............8
Figure S4. MIBiG BGCs with high similarity to orphan *Salinispora* BGCs.……………………................9
Figure S5. MIBiG enediyne BGCs with high similarity to orphan *Salinispora* BGCs.……..……............10
Figure S6. MIBiG BGCs with low yet meaningful similarity to orphan *Salinispora* BGCs………...........11
Figure S7. NMDS plots of GCF distribution among *S. arenicola* strains…..………………….................12
Figure S8. Distance matrix heatmap plot of GCF distribution among *S. arenicola* strains……………...13
Figure S9. MS spectra and molecular formulae of azinothricin analogs……………………..……………14
Figure S10. HPLC-UV-ELSD of *S. oceanensis* CNZ-875 ……….……...…………………..................15
Figure S11. MS and UV/Vis spectra of fridamycin E………..……………………………...................15
Figure S12. BGC of fridamycin and known angucycline BGCs…..…………………………….............16
Figure S13. Candidate fridamycin BGCs across *Salinispora*……..……………………………..…………17

Genome assemblies. Assembly steps were: raw reads QC and trimming (bbduk: 38.93 run with options --minlength 35; --trimq 10, sourceforge.net/projects/bbmap/); fastq-scan: 0.4.4 (https://github.com/rpetit3/fastq-scan); fastqc: 0.11.9 (https://github.com/s-andrews/FastQC); lighter: 1.1.2; mash: '2.3'; mccortex: 0.0.3-610-g400c0e3; sourmash: 4.2.2; genome assembly (shovill: 1.1.0, which included tools: seqtk, bbmap, flash: 1.2.11, spades.py: 3.15.3 (run with --isolate option) (https://github.com/tseemann/shovill), bwa: 0.7.17-r1188, samtools: '1.12', pilon: '1.24' (1); genome assembly QC (checkm: 1.1.3; quast: 5.0.2 (2)); and genome annotation (prokka: 1.14.6 (--compliant; --centre JensenSIO) (3). Three different assembly methods were compared, including spades.py: 3.15.3 (4), skesa: 2.4.0 (5), and velvetg: 1.2.10 (6). The SPades assembly resulted in the most contiguous assembly with the least number of contigs, as assessed by comparing assembly and coverage settings and visualizing resulting assemblies with Bandage (7).

For the MiSeq samples, no coverage correction was needed as genome assemblies averaged ~48x (median depth ~20x) when run at 100x maximum coverage of reads. For NovaSeq samples, 600x coverage resulted in the most contiguous and complete genome assembly. We further investigated the high coverage assembly method where SNPs and indels were only corrected with Pilon (1) if the coverage of reads was 0.25% of the coverage on contigs >10,000 bp; the SPades assembly used both R1, R2, and the additional dataset of overlapping merged reads assembled with Flash (8). More coverage did not increase the error correction rate, only an increase in the longest contig and average N50 value. Manual inspection of the genome assemblies revealed that the 600x coverage assemblies contained less orphaned nodes/contigs and dead ends.

antiSMASH parameters. antismash --genefinding-tool prodigal, --cb-general, --cb-knownclusters, --cb-subclusters, --asf, --pfam2go, --rre, --cc-mibig, --tigrfam, --smcog-trees.

BiG-SCAPE parameters. --mibig --cutoffs 0.3 (0.4 and 0.6) --mix --no_classify.

MZmine parameters. Mass detection (MS^1 noise level set to 1E3), chromatogram builder (0.1 min, 1E5 min height, 0.0 m/z tolerance, 20 ppm), chromatogram deconvolution (baseline cut-off, no MS^2 pairing), isotope peak grouper (0.0 m/z tolerance, 20 ppm, 0.1 min t_R tolerance, 3 maximum charge), and join aligner (0.0 m/z tolerance, 20 ppm, 0.1 min t_R tolerance, 75 weight for m/z, 25 weight for t_R).

References

1. Walker BJ, Abeel T, Shea T, Priest M, Abouelliel A, Sakthikumar S, Cuomo CA, Zeng Q, Wortman J, Young SK. 2014. Pilon: an integrated tool for comprehensive microbial variant detection and genome assembly improvement. PloS One 9:e112963.
2. Gurevich A, Saveliev V, Vyahhi N, Tesler G. 2013. QUAST: quality assessment tool for genome assemblies. Bioinformatics 29:1072-1075.
3. Seemann T. 2014. Prokka: rapid prokaryotic genome annotation. Bioinformatics 30:2068-2069.
4. Bankevich A, Nurk S, Antipov D, Gurevich AA, Dvorkin M, Kulikov AS, Lesin VM, Nikolenko SI, Pham S, Prjibelski AD. 2012. SPAdes: a new genome assembly algorithm and its applications to single-cell sequencing. J Comp Biol 19:455-477.
5. Souvorov A, Agarwala R, Lipman DJ. 2018. SKESA: strategic k-mer extension for scrupulous assemblies. Genome Biology 19:153.
6. Zerbino DR, Birney E. 2008. Velvet: Algorithms for de novo short read assembly using de Bruijn graphs. Genome Res 18:821-829.
7. Wick RR, Schultz MB, Zobel J, Holt KE. 2015. Bandage: interactive visualization of de novo genome assemblies. Bioinformatics 31:3350-3352.
8. Magoč T, Salzberg SL. 2011. FLASH: fast length adjustment of short reads to improve genome assemblies. Bioinformatics 27:2957-2963.

Figure S1. Morphologies of microscale *Salinispora* strains grown on agar media. One representative strain from each sub-quadrat is shown. Pictures were taken around the same time of growth at 1-3 weeks.

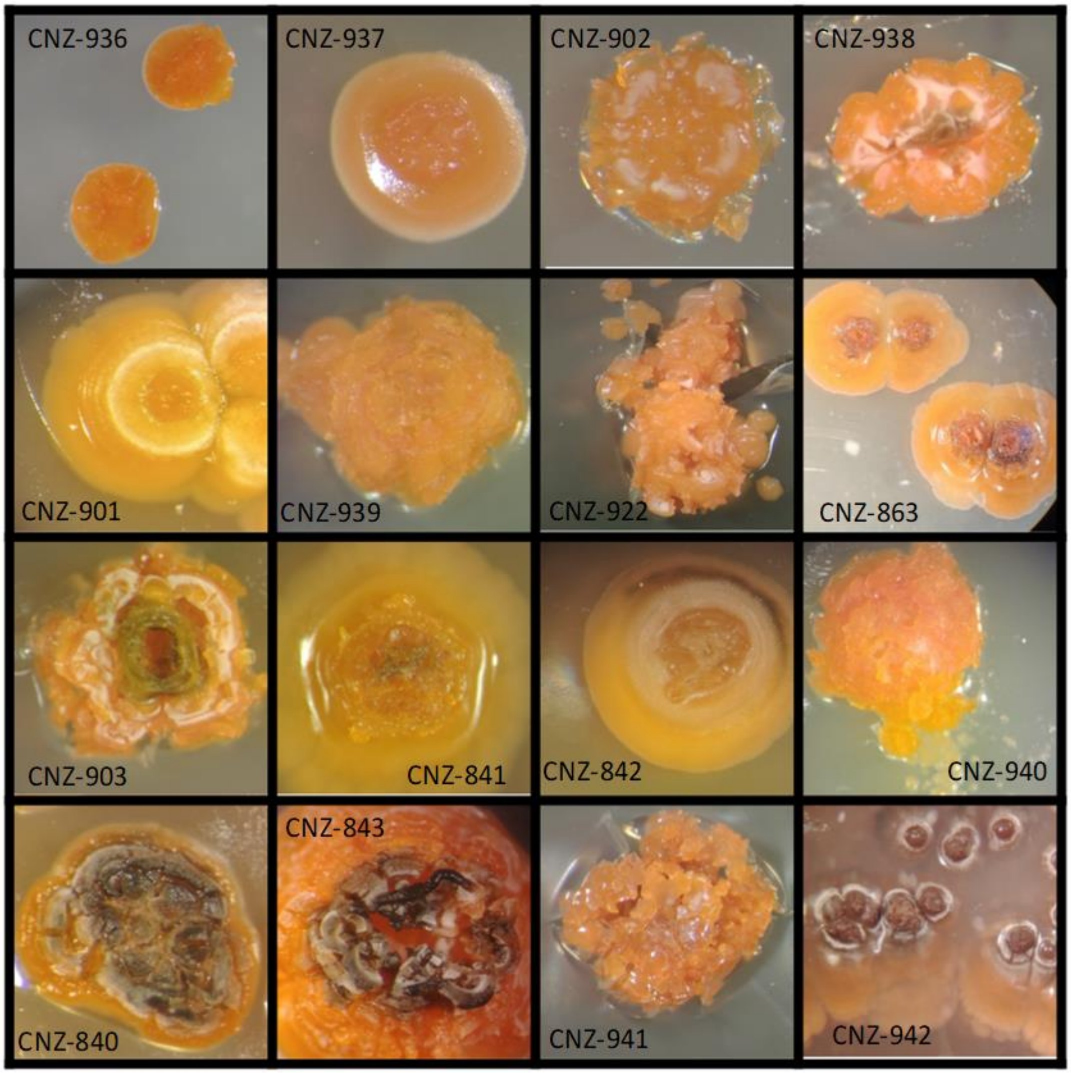

Table S1. Microscale genome statistics and accession numbers.

n°	Strain	Genome length (Mb)	Number of contigs	Longest contig (Mb)	Gene count	GC content (%)	NCBI Biosample Accession number	NCBI Genome accession number
1	S. arenicola CNZ840	5.68	55	0.713	5064	69.6	SAMN44795772	JBJKBF000000000
2	S. arenicola CNZ841	5.60	55	0.727	5035	69.6	SAMN44795773	JBJKBE000000000
3	S. arenicola CNZ842	5.50	51	0.654	4974	69.6	SAMN44795774	JBJKBD000000000
4	S. arenicola CNZ843	5.71	42	0.781	5186	69.6	SAMN44795775	JBJKBC000000000
5	S. arenicola CNZ844	5.60	96	0.392	4972	69.6	SAMN44795776	JBJKBB000000000
6	S. arenicola CNZ846	5.70	105	0.510	5064	69.6	SAMN44795777	JBJKBA000000000
7	S. arenicola CNZ848	5.60	90	0.566	4977	69.6	SAMN44795778	JBJKAZ000000000
8	S. arenicola CNZ850	5.61	103	0.319	4981	69.6	SAMN44795779	JBJKAY000000000
9	S. arenicola CNZ854	5.72	111	0.493	5080	69.6	SAMN44795780	JBJKAX000000000
10	S. arenicola CNZ856	5.64	81	0.415	5099	69.6	SAMN44795781	JBJKAW000000000
11	S. arenicola CNZ858	5.75	113	0.468	5138	69.7	SAMN44795782	JBJKAV000000000
12	S. arenicola CNZ863	5.77	50	0.832	5236	69.6	SAMN44795783	JBJKAU000000000
13	S. arenicola CNZ864	5.51	86	0.346	4957	69.6	SAMN44795784	JBJKAT000000000
14	S. arenicola CNZ871	5.90	86	0.338	5255	69.6	SAMN44795786	JBJKAR000000000
15	S. arenicola CNZ872	5.68	96	0.390	5083	69.6	SAMN44795787	JBJKAQ000000000
16	S. arenicola CNZ877	5.50	75	0.531	4951	69.7	SAMN44795789	JBJKAO000000000
17	S. arenicola CNZ878	5.51	71	0.580	4959	69.7	SAMN44795790	JBJKAN000000000
18	S. arenicola CNZ880	5.81	101	0.412	5153	69.6	SAMN44795791	JBJKAM000000000
19	S. arenicola CNZ883	5.65	79	0.389	5091	69.5	SAMN44795792	JBJKAL000000000
20	S. arenicola CNZ886	5.57	78	0.394	5025	69.6	SAMN44795793	JBJKAK000000000
21	S. arenicola CNZ891	5.68	91	0.544	5158	69.6	SAMN44795794	JBJKAJ000000000
22	S. arenicola CNZ894	5.62	80	0.354	5042	69.6	SAMN44795795	JBJKAI000000000
23	S. arenicola CNZ896	5.50	118	0.316	4930	69.6	SAMN44795796	JBJKAH000000000
24	S. arenicola CNZ901	5.69	51	0.832	5173	69.6	SAMN44795797	JBJKAG000000000
25	S. arenicola CNZ902	5.49	54	0.403	4894	69.6	SAMN44795798	JBJKAF000000000
26	S. arenicola CNZ903	5.50	49	0.522	4921	69.6	SAMN44795799	JBJKAE000000000
27	S. arenicola CNZ904	5.60	100	0.338	4951	69.6	SAMN44795800	JBJKAD000000000
28	S. arenicola CNZ912	5.63	88	0.500	5007	69.7	SAMN44795801	JBJKAC000000000
29	S. arenicola CNZ915	5.69	77	0.556	5062	69.6	SAMN44795802	JBJKAB000000000
30	S. arenicola CNZ916	5.52	119	0.345	4984	69.6	SAMN44795803	JBJKAA000000000
31	S. arenicola CNZ918	5.78	98	0.382	5244	69.5	SAMN44795804	JBJJZZ000000000
32	S. arenicola CNZ920	5.72	111	0.493	5067	69.6	SAMN44795805	JBJJZY000000000
33	S. arenicola CNZ922	5.75	49	0.519	5200	69.6	SAMN44795806	JBJJZX000000000
34	S. arenicola CNZ924	5.60	88	0.389	4993	69.6	SAMN44795807	JBJJZW000000000
35	S. arenicola CNZ928	5.66	95	0.471	5129	69.6	SAMN44795808	JBJJZV000000000
36	S. arenicola CNZ934	5.52	97	0.446	4993	69.6	SAMN44795809	JBJJZU000000000
37	S. arenicola CNZ935	5.70	98	0.545	5137	69.6	SAMN44795810	JBJJZT000000000
38	S. arenicola CNZ936	5.63	36	1.162	5011	69.7	SAMN44795811	JBJJZS000000000
39	S. arenicola CNZ937	5.72	60	0.662	5084	69.6	SAMN44795812	JBJJZR000000000
40	S. arenicola CNZ938	5.91	60	0.565	5293	69.5	SAMN44795813	JBJJZQ000000000
41	S. arenicola CNZ939	5.62	50	0.696	5066	69.6	SAMN44795814	JBJJZP000000000

42	S. arenicola CNZ940	5.60	68	0.547	4929	69.6	SAMN44795815	JBJJZO000000000
43	S. arenicola CNZ941	5.75	49	1.105	5088	69.6	SAMN44795816	JBJJZN000000000
44	S. arenicola CNZ942	5.68	48	0.612	5034	69.6	SAMN44795817	JBJJZM000000000
45	S. arenicola CNZ944	5.62	87	0.393	5033	69.7	SAMN44795818	JBJJZL000000000
46	S. arenicola CNZ946	5.49	87	0.439	4955	69.6	SAMN44795819	JBJJZK000000000
47	S. arenicola CNZ947	5.54	85	0.416	5029	69.6	SAMN44795820	JBJJZJ000000000
48	S. arenicola CNZ948	5.47	103	0.316	4908	69.6	SAMN44795821	JBJJZI000000000
49	S. arenicola CNZ950	5.55	78	0.458	5044	69.6	SAMN44795822	JBJJZH000000000
50	S. arenicola CNZ951	5.67	96	0.560	5126	69.6	SAMN44795823	JBJJZG000000000
51	S. arenicola CNZ952	5.48	93	0.382	4904	69.6	SAMN44795824	JBJJZF000000000
52	S. arenicola CNZ953	5.61	98	0.322	4973	69.6	SAMN44795825	JBJJZE000000000
53	S. arenicola CNZ956	5.66	78	0.430	5109	69.6	SAMN44795826	JBJJZD000000000
54	S. arenicola CNZ957	5.60	119	0.412	5009	69.7	SAMN44795827	JBJJZC000000000
55	S. arenicola CNZ959	5.54	87	0.412	4941	69.7	SAMN44795828	JBJJZB000000000
56	S. arenicola CNZ960	5.71	109	0.356	5111	69.6	SAMN44795829	JBJJZA000000000
57	S. arenicola CNZ963	5.70	93	0.509	5077	69.6	SAMN44795830	JBJJYZ000000000
58	S. arenicola CNZ965	5.52	108	0.431	4966	69.6	SAMN44795831	JBJJYY000000000
59	S. arenicola CNZ970	5.47	82	0.460	4946	69.6	SAMN44795833	JBJJYW000000000
60	S. arenicola CNZ971	5.71	117	0.361	5061	69.6	SAMN44795834	JBJJYV000000000
61	S. arenicola CNZ972	5.59	79	0.458	5072	69.6	SAMN44795835	JBJJYU000000000
62	S. arenicola CNZ974	5.69	77	0.454	5042	69.6	SAMN44795836	JBJJYT000000000
63	S. arenicola CNZ975	5.66	83	0.426	5109	69.6	SAMN44795837	JBJJYS000000000
64	S. arenicola CNZ984	5.76	82	0.392	5133	69.6	SAMN44795838	JBJJYR000000000
65	S. arenicola CNZ989	5.49	88	0.332	4876	69.6	SAMN44795839	JBJJYQ000000000
66	S. arenicola CNZ991	5.66	94	0.330	5041	69.7	SAMN44795840	JBJJYP000000000
67	S. arenicola CNZ994	5.62	97	0.384	5078	69.6	SAMN44795841	JBJJYO000000000
68	S. arenicola CNZ995	5.64	82	0.423	5062	69.6	SAMN44795842	JBJJYN000000000
69	S. arenicola CNZ997	5.74	105	0.542	5141	69.6	SAMN44795843	JBJJYM000000000
70	S. arenicola SNA004	5.51	83	0.446	4972	69.6	SAMN44795844	JBJJYL000000000
71	S. arenicola SNA006	5.49	73	0.402	4881	69.7	SAMN44795845	JBJJYK000000000
72	S. arenicola SNA010	5.66	98	0.368	5053	69.6	SAMN44795846	JBJJYJ000000000
73	S. arenicola SNA012	5.63	106	0.545	4994	69.6	SAMN44795868	JBJJYI000000000
74	S. arenicola SNA013	5.59	87	0.527	5052	69.6	SAMN44795847	JBJJYH000000000
75	S. arenicola SNA015	5.65	92	0.393	5032	69.6	SAMN44795848	JBJJYG000000000
76	S. arenicola SNA017	5.56	73	0.392	4948	69.6	SAMN44795849	JBJJYF000000000
77	S. arenicola SNA019	5.59	84	0.522	5023	69.7	SAMN44795850	JBJJYE000000000
78	S. arenicola SNA023	5.60	75	0.446	5041	69.6	SAMN44795851	JBJJYD000000000
79	S. arenicola SNA024	5.73	78	0.421	5088	69.6	SAMN44795852	JBJJYC000000000
80	S. arenicola SNA030	5.59	82	0.485	5008	69.6	SAMN44795853	JBJJYB000000000
81	S. arenicola SNA032	5.75	107	0.365	5244	69.5	SAMN44795869	JBJJYA000000000
82	S. arenicola SNA034	5.51	84	0.475	5015	69.6	SAMN44795854	JBJJXZ000000000
83	S. arenicola SNA036	5.70	89	0.394	5186	69.6	SAMN44795855	JBJJXY000000000
84	S. arenicola SNA039	5.66	100	0.426	5130	69.5	SAMN44795856	JBJJXX000000000
85	S. arenicola SNA040	5.51	93	0.412	4953	69.6	SAMN44795870	JBJJXW000000000
86	S. arenicola SNA041	5.62	99	0.318	5095	69.6	SAMN44795857	JBJJXV000000000
87	S. arenicola SNA045	5.65	104	0.550	5056	69.6	SAMN44795858	JBJJXU000000000

88	S. arenicola SNA046	5.78	106	0.480	5171	69.6	SAMN44795859	JBJJXT000000000
89	S. arenicola SNA047	5.68	95	0.356	5074	69.6	SAMN44795860	JBJJXS000000000
90	S. arenicola SNA051	5.85	87	0.409	5209	69.6	SAMN44795861	JBJJXR000000000
91	S. arenicola SNA055	5.74	109	0.480	5116	69.7	SAMN44795862	JBJJXQ000000000
92	S. arenicola SNA056	5.70	137	0.342	5128	69.6	SAMN44795863	JBJJXP000000000
93	S. arenicola SNA057	5.65	77	0.436	5119	69.6	SAMN44795864	JBJJXO000000000
94	S. arenicola SNA058	5.67	119	0.416	5162	69.6	SAMN44795865	JBJJXN000000000
95	S. arenicola SNA059	5.87	124	0.389	5304	69.6	SAMN44795866	JBJJXM000000000
96	S. arenicola SNA064	5.70	107	0.299	5145	69.6	SAMN44795867	JBJJXL000000000
97	S. oceanensis CNZ875	5.37	101	0.329	5027	69.7	SAMN44795788	JBJKAP000000000
98	S. oceanensis CNZ966	5.53	105	0.446	5139	69.6	SAMN44795832	JBJJYX000000000
99	S. pacifica CNZ865	5.56	134	0.395	5070	69.8	SAMN44795785	JBJKAS000000000

Figure S2. Phylogeny of microscale and global *Salinispora* strains based on 324 conserved single-copy marker genes. The maximum likelihood tree was calculated with a PROTCATLG model of evolution with 100 bootstraps in RAxML. Color bars indicate 1) *Salinispora* species, 2) *S. arenicola* 99% ANI population, and 3) strain isolation location.

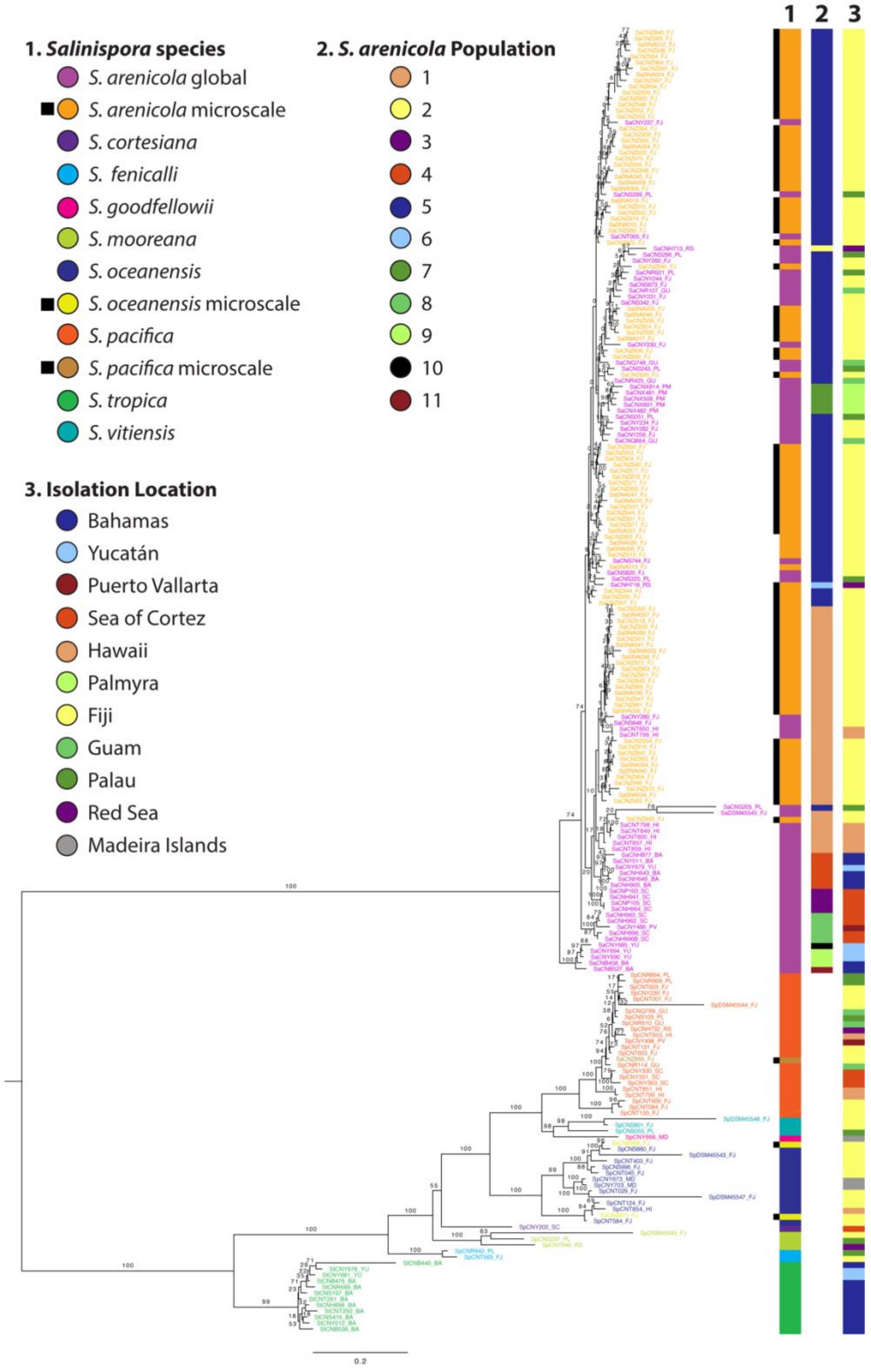

Figure S3. Manual assessment of BiG-SCAPE GCFs from microscale *Salinispora arenicola* strains.

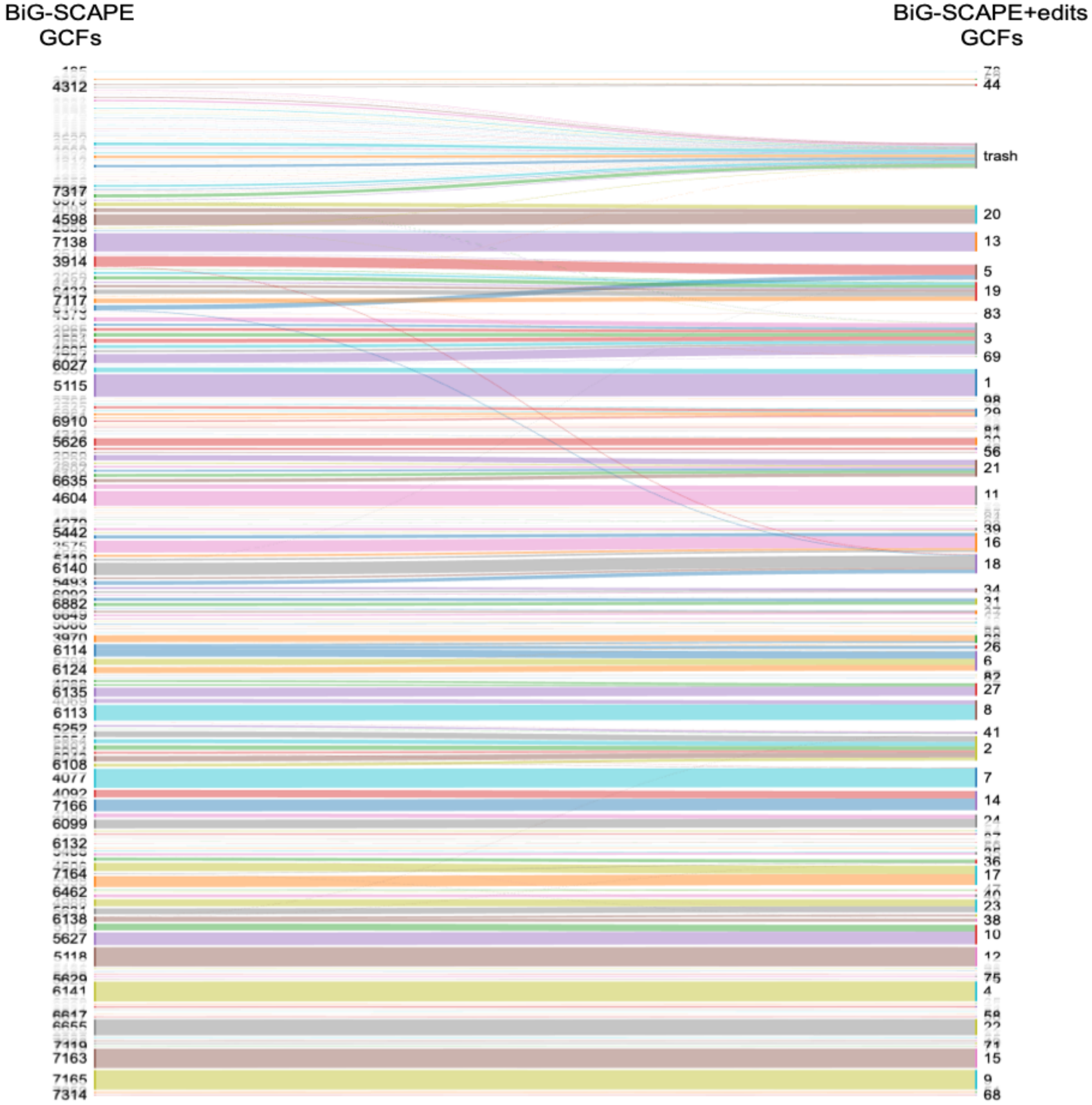

Diagram shows the transition from the initial 204 GCFs identified with BiG-SCAPE ("BiG-SCAPE GCFs", left) to the final 100 GCFs ("BiG-SCAPE+edits", right). Forty-four GCFs were removed (trash) because their contigs were deemed largely incomplete. The remaining 164 GCFs were consolidated into 91 GCFs. These are represented in the figure as converging lines going from left to right. Nine GCFs were added because they were missed by antiSMASH. Diagram built with https://sankeymatic.com/build

Figure S4. MIBiG BGCs with high similarity to orphan microscale *Salinispora* BGCs.

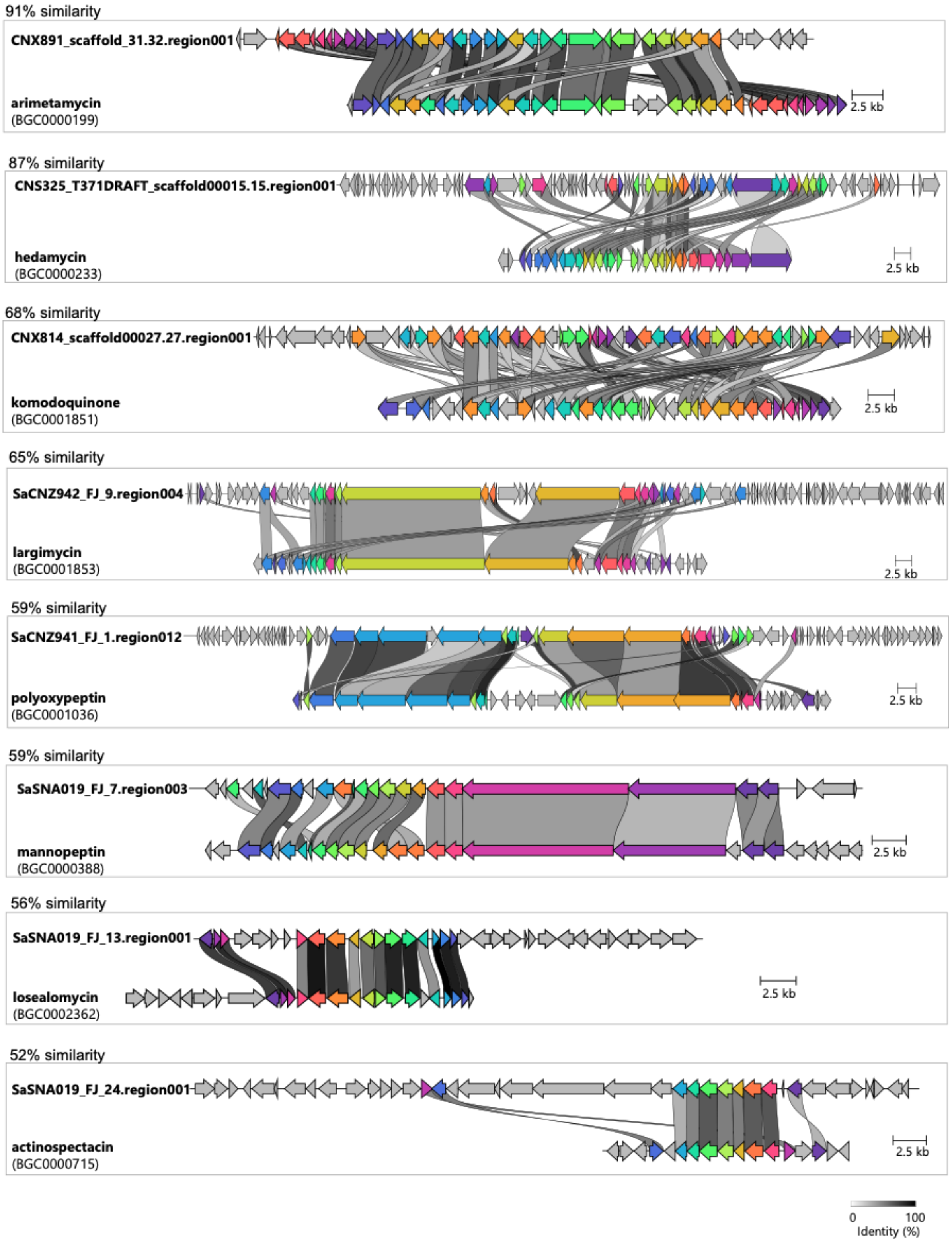

Figure S5. MIBiG enediyne BGCs with similarity to orphan microscale *Salinispora* BGCs. BGCs with enediyne KSs are indicated.

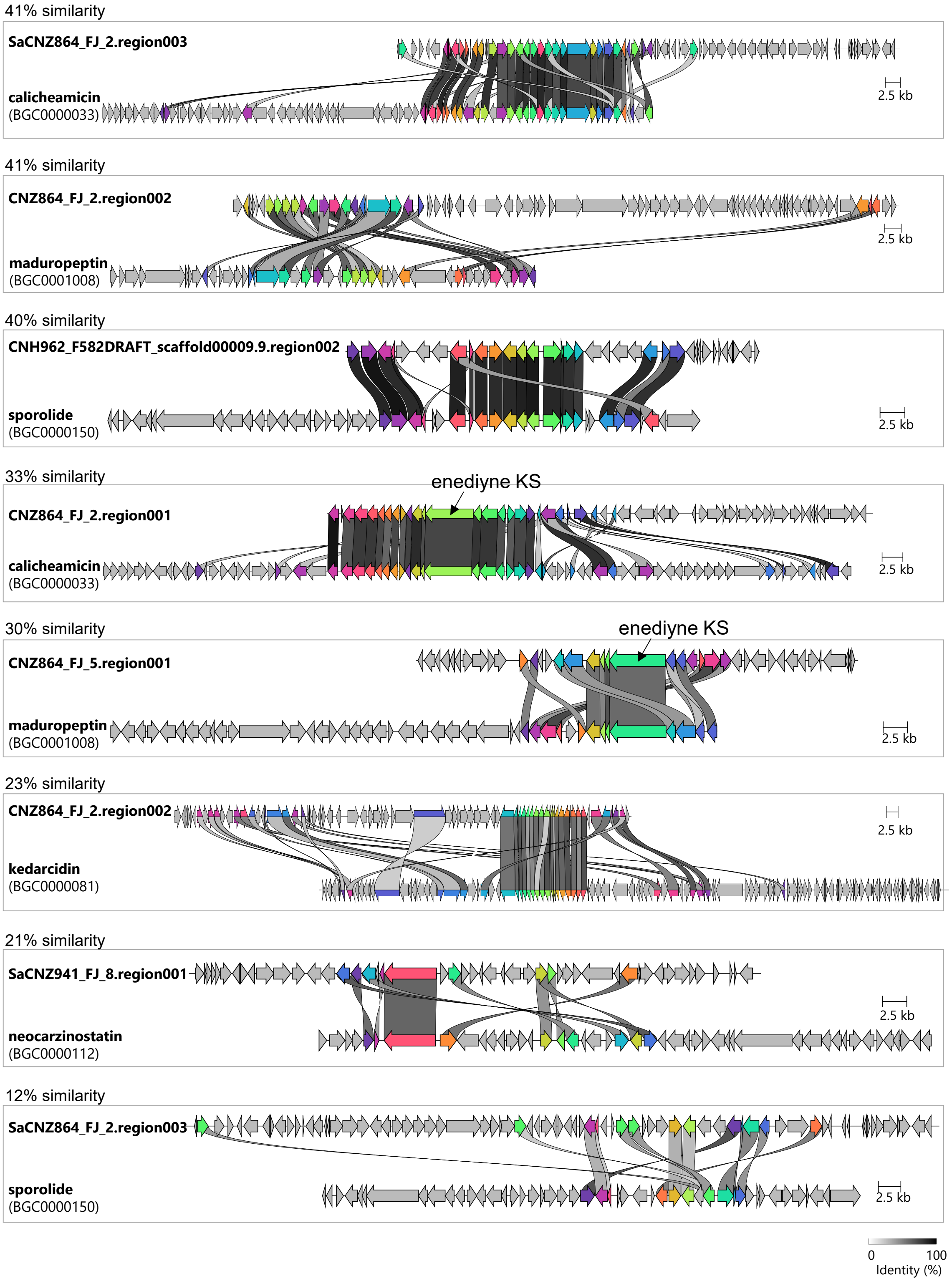

Figure S6. MIBiG BGCs with low yet meaningful similarity to orphan microscale *Salinispora* BGCs.

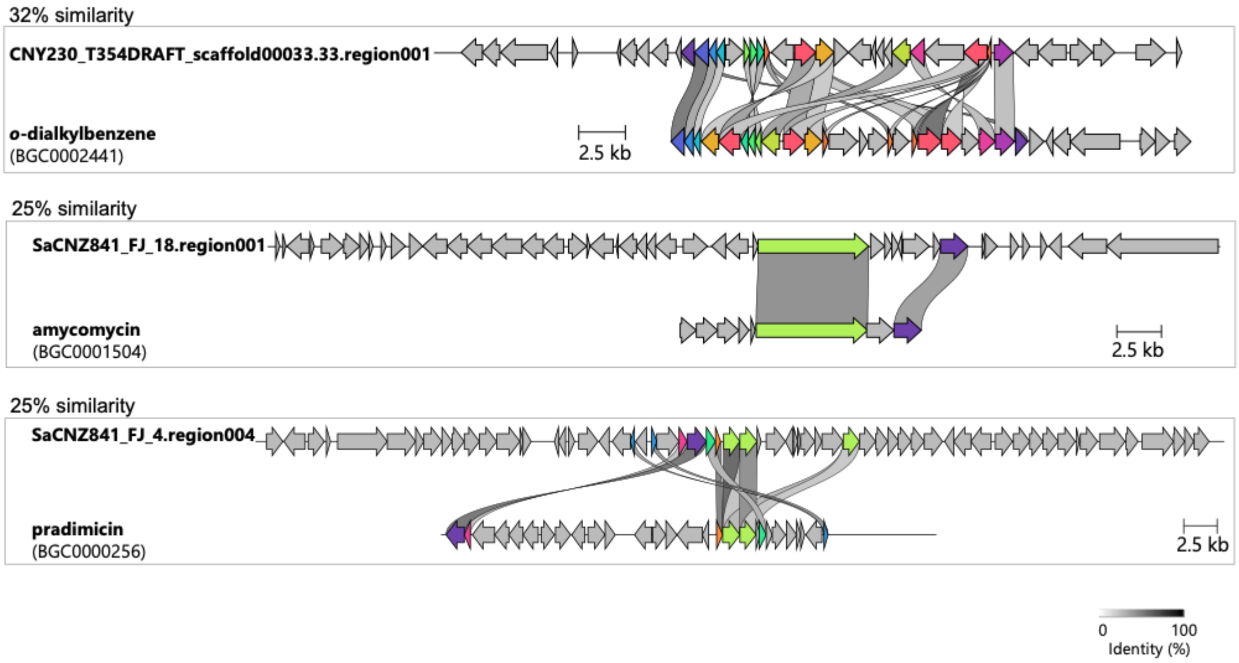

Figure S7. Non-metric multidimension scaling (NMDS) plots of GCF distribution among microscale and global *S. arenicola* strains. Top: Each dot represents a genome colored by population. Bottom: Each dot represents a genome colored by location of origin.

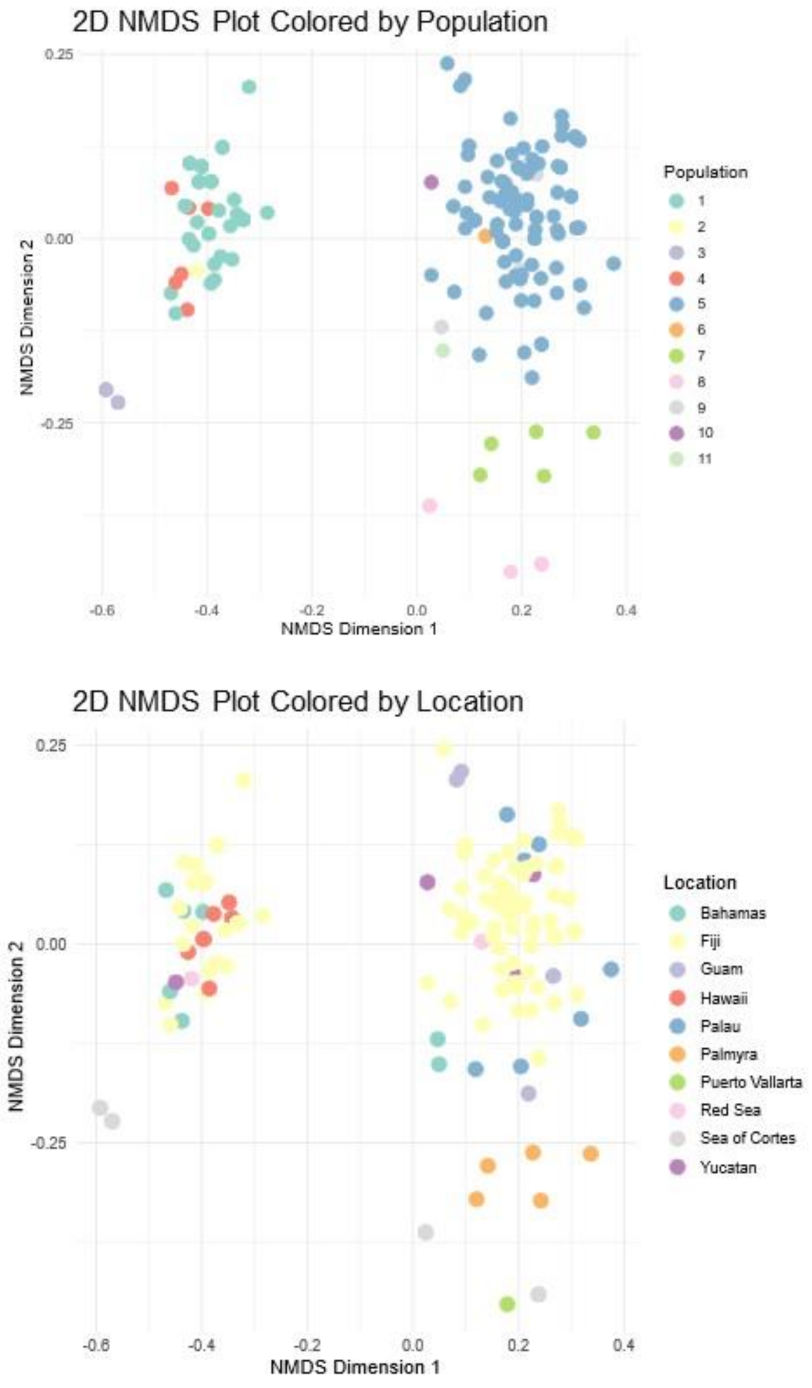

Figure S8. Distance matrix heatmap plot of GCF distribution among global and microscale *S. arenicola* strains.

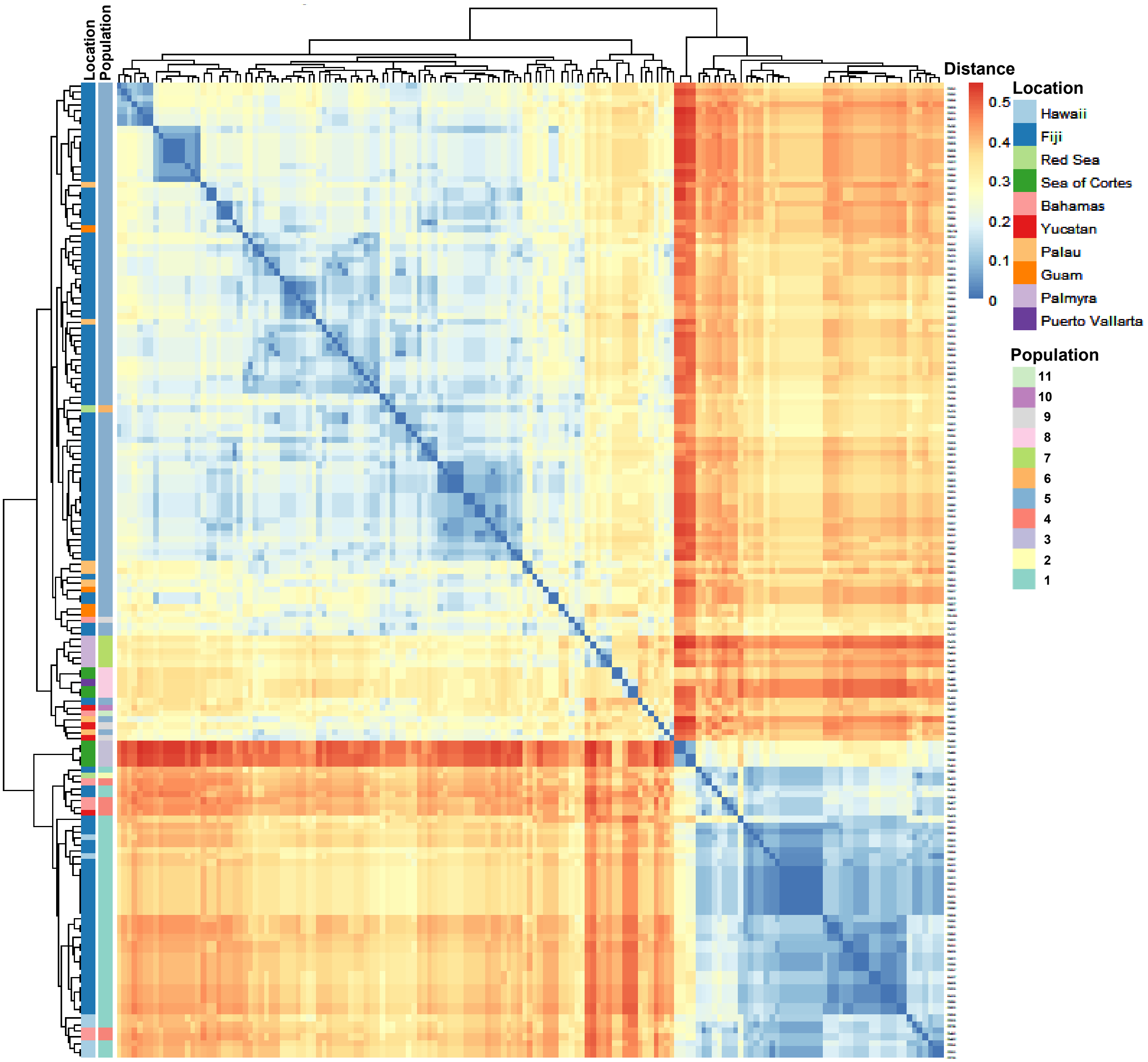

Figure S9. MS spectra (in positive and negative mode ionization) and molecular formulae of azinothricin analogs produced by *S. arenicola* CNZ-941.

$C_{47}H_{80}N_8O_{18}$ – new putative azinothricin analog

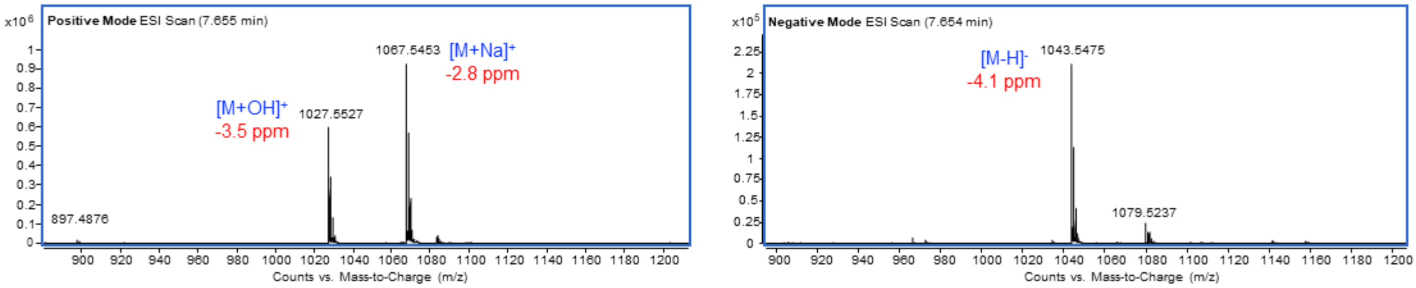

$C_{38}H_{66}N_8O_{14}$ – new putative azinothricin analog

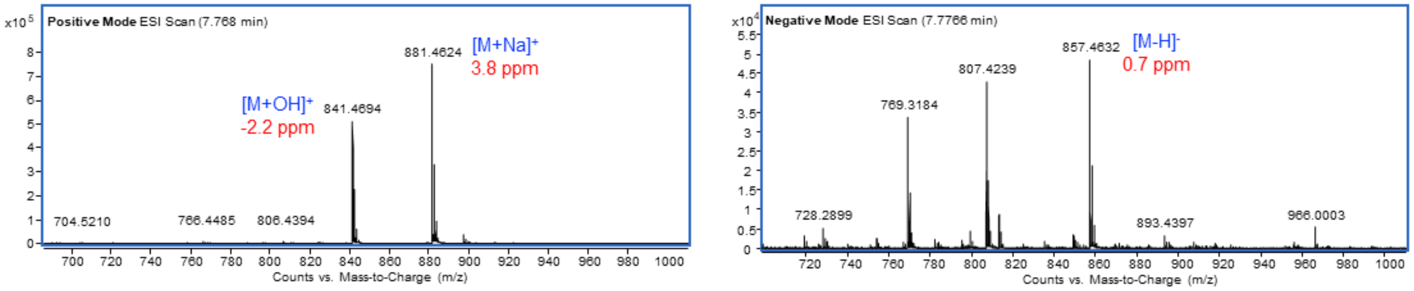

$C_{35}H_{60}N_8O_{12}$ – same molecular formula as azinothricin analog dentigerumycin G

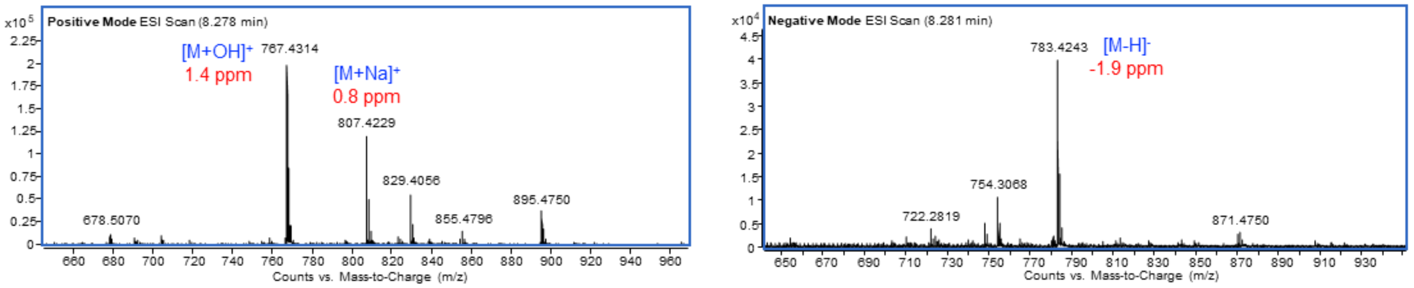

Figure S10. HPLC-UV-ELSD of *S. oceanensis* CNZ-875 extract showing fridamycin E production.

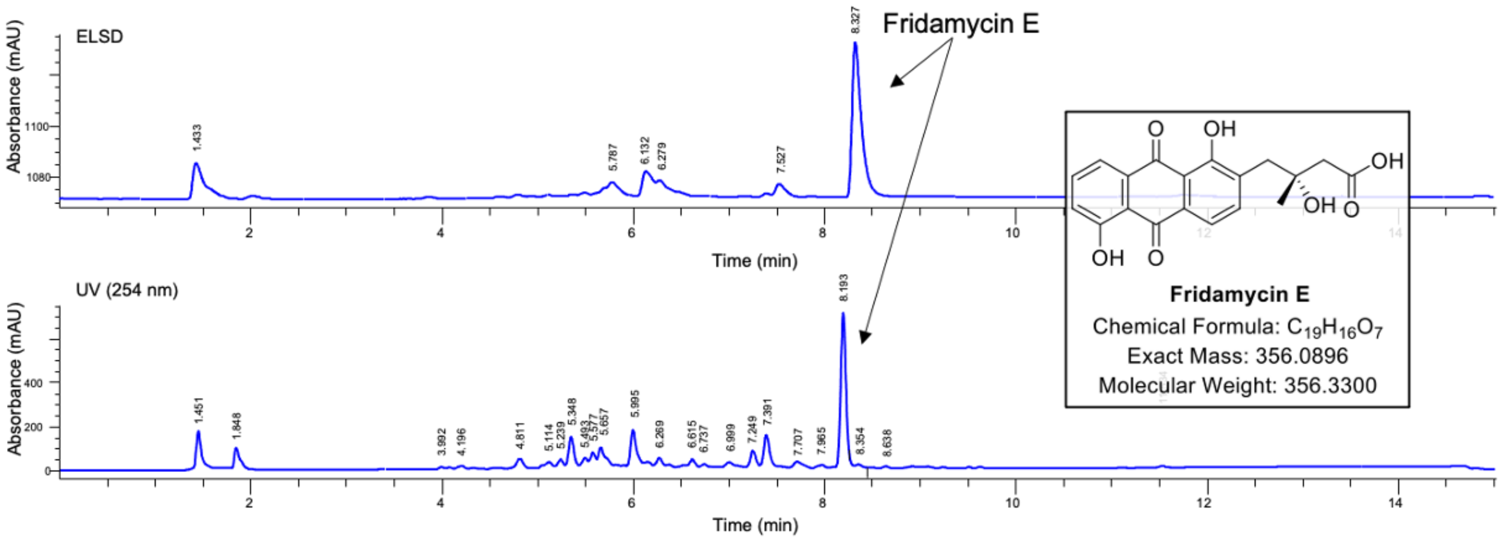

Figure S11. HRMS and UV/vis spectra of fridamycin E in *S. oceanensis* CNZ-875 extract. UV/vis spectrum for fridamycin E reference standard is also shown (red curve).

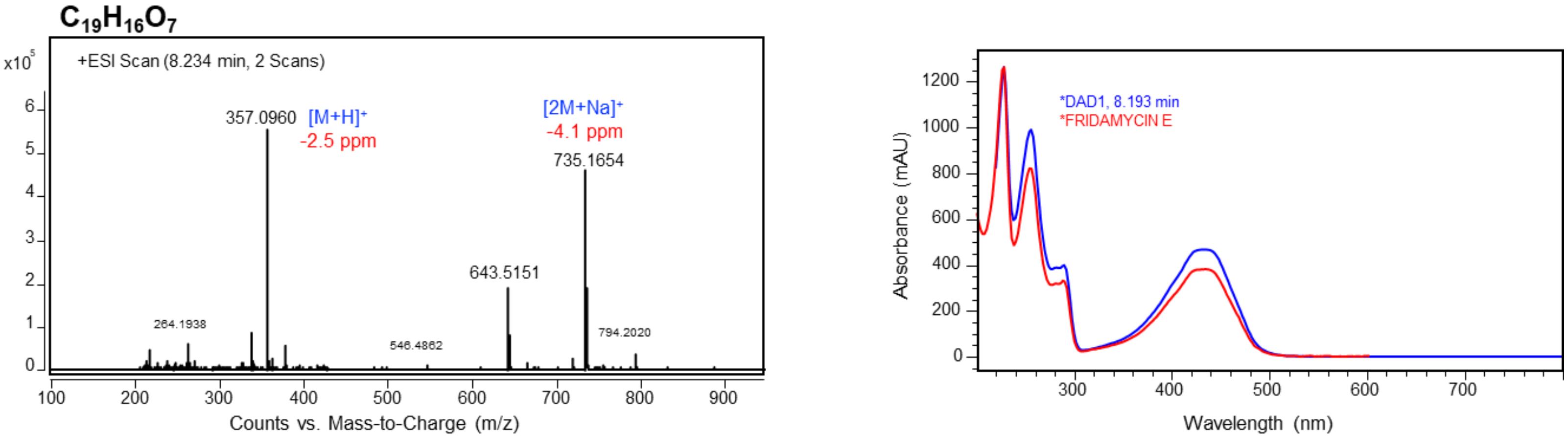

Figure S12. Candidate fridamycin biosynthetic gene cluster in *S. oceanensis* CNZ-875 and known angucycline BGCs. Synteny plot of two type II PKS BGCs in *S. oceanensis* CNZ-875 (black labels) and known angucyline BGCs (red labels). Percent identities between genes is indicated by the color of the line that connects genes (see grayscale legend). The candidate fridamycin BGC is indicated with a star. The genes involved in angucycline biosynthesis are numbered (1-8) and their putative functions are indicated (below the plot). Known angucycline BGCs include their MIBiG repository or NCBI accession numbers.

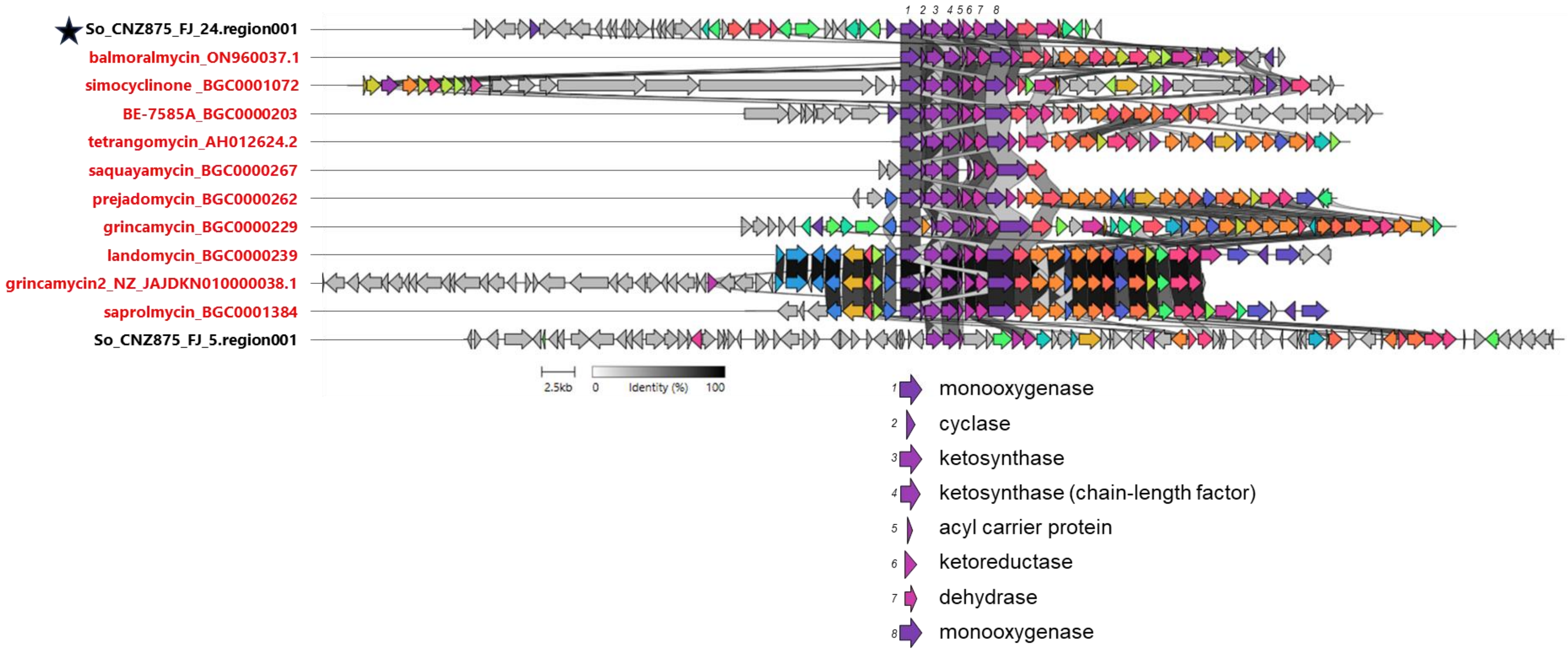

Figure S13. Candidate fridamycin biosynthetic gene clusters (BGCs) in *S. oceanensis* and *S. fenicalii*. Synteny plot of the five candidate fridamycin BGCs identified in 217 *Salinispora* strains. Percent identities between genes is indicated by the color of the line that connects genes (see grayscale legend). The genes involved in angucycline biosynthesis are numbered (1-8) and their putative functions are indicated (below the plot).

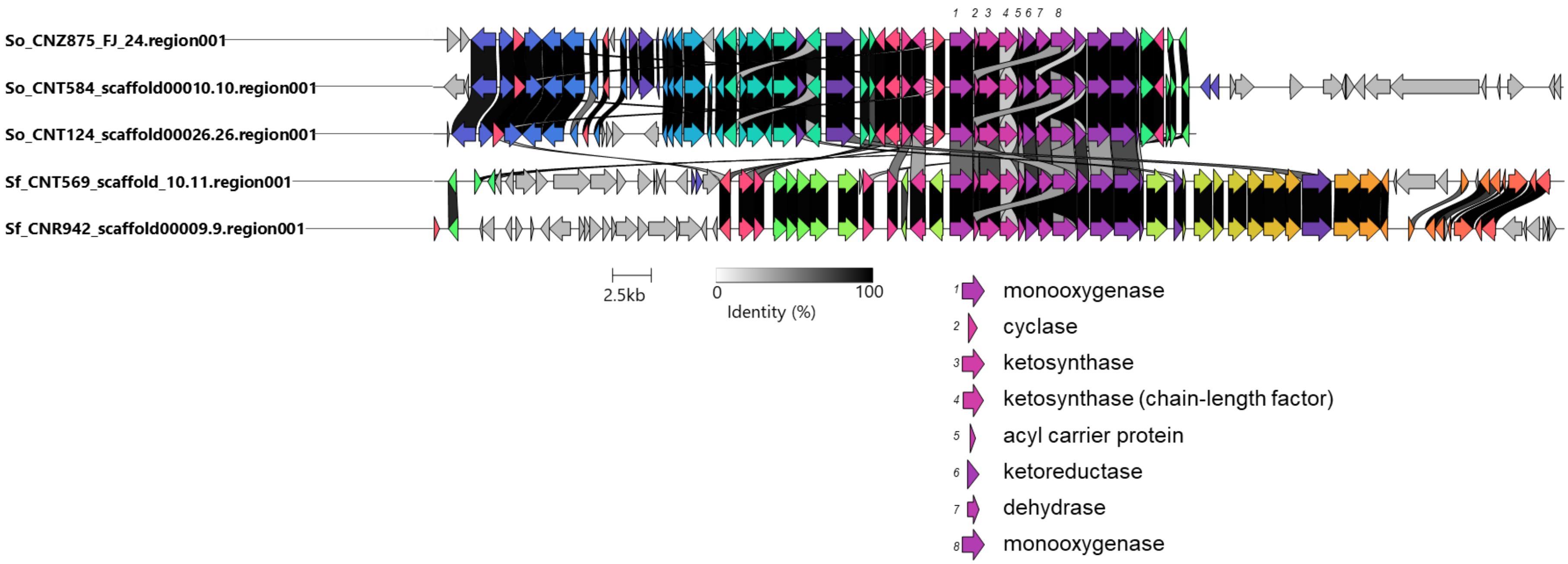

Supplement: Supplemental material — Supplemental methods, Table S1, and Figures S1 to S13. [file aem.02171-25-s0004.pdf]
